# Light-Responsive Supramolecular Nanotubes-Based Chiral Plasmonic Assemblies

**DOI:** 10.1021/acsnano.2c10955

**Published:** 2023-03-10

**Authors:** Agnieszka Jedrych, Mateusz Pawlak, Ewa Gorecka, Wiktor Lewandowski, Michal Maksymilian Wojcik

**Affiliations:** Faculty of Chemistry, University of Warsaw, 1 Pasteur Street, 02-093 Warsaw, Poland

**Keywords:** organic nanotubes, liquid crystals, nanocomposites, plasmonic nanoparticles, reversibly reconfigurable assembly, supramolecular self-assembly, photoswitchability

## Abstract

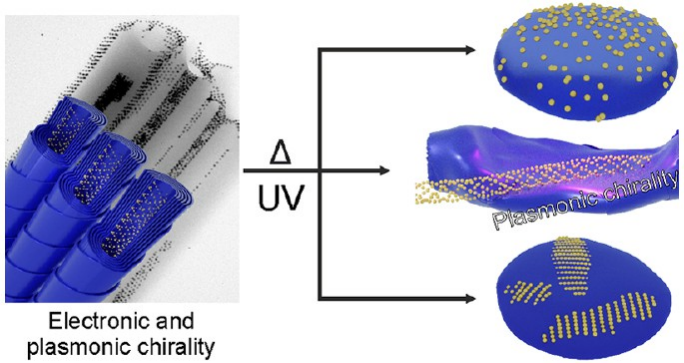

We describe the fabrication of dual-responsive (thermo/light) chiral plasmonic films. The idea is based on using photoswitchable achiral liquid crystal (LCs) forming chiral nanotubes for templating helical assemblies of Au NPs. Circular dichroism spectroscopy (CD) confirms chiroptical properties coming from the arrangement of organic and inorganic components, with up to 0.2 dissymmetry factor (g-factor). Upon exposure to UV light, organic molecules isomerize, resulting in controlled melting of organic nanotubes and/or inorganic nanohelices. The process can be reversed using visible light and further modified by varying the temperature, offering a control of chiroptical response of the composite material. These properties can play a key role in the future development of chiral plasmonics, metamaterials, and optoelectronic devices.

Modern optoelectronic technology requires maximum miniaturization without compromising on quality of optical response, precise control of nanoscopic organization, and an effective response to stimuli delivered preferably without direct contact.^1−3^ Thus, e.g., light-responsive polymers,^4,5^ DNA assemblies,^6,7^ cavities,^8^ and photonic crystals^9^ were considered as crucial components of optoelectronic devices. In addition to these, liquid crystals are particularly attractive building blocks as they may exhibit hierarchical order and high levels of reactivity to external stimuli. Additionally, they may support relatively strong chiroptical properties in thin films, which may be useful for technologies relying on the selective absorption of circularly polarized light.^10−12^

From a design perspective, encoding chirality and temperature responsiveness of thermotropic LCs is achieved through selection of aromatic cores and alkyl chains, while light responsiveness is usually achieved by irradiating compounds equipped with an azo unit, a moiety that can be switched between the Z and E isomers. Azo molecules are typically used as reactive dopants in LC matrices,^13−16^ but they are more desirable to combine mesogenic properties, hierarchical order, chiroptical properties, and photoswitchability in a single compound.^17^ This ambitious goal was achieved by Takezoe et al., who showed that the azo dimer, 12OAzo5AzoO12, forms helical nanoribbons organized into homochiral domains upon irradiating with circularly polarized light^18^ or by mixing with a compound exhibiting the N phase in twisted nematic cell,^19^ sometimes a centimeter large. Similarly, Yoon et al.^20,21^ showed that UV irradiation was able to generate oriented arrays of such azo dimer helical nanofilaments. Despite these benefits, the overall intrinsic limitations of chiral organic materials motivate combining chiral, organic hosts with inorganic nanoparticles (NPs) into composite assemblies.^22−30^ Such composite materials were shown to exhibit strong light–matter interactions and tunable spectral range of chiral responses. However, remote switching of chiroptical thin-film composite materials is challenging.^31−33^

We hypothesized that the combination of metallic NPs with light-responsive, chiral LC material could overcome these barriers and offer access to multifunctional optoelectronic materials with chiroptical properties.^34^ The combination of LC properties with metallic^35−38^ and semiconductor^39,40^ NPs allowed for the observation of a number of physical effects that are encoded in and enhanced by the proper ordering of NPs.^41^ Since these properties are dependent on NPs order,^42^ and NPs packing is dependent on LC, it is possible to control LC/NPs composite materials using factors such as temperature or UV and Vis light irradiation.^43,44^ Beyond remote control, preliminary studies have shown that composites obtained by doping morphologically chiral LC with Au NPs offer possibilities for the construction of soft and chiral functional systems.^35,36^ However, achieving highly organized chiral assemblies of NPs with properties controlled remotely, and via multiple stimuli, all in one system, has not been achieved yet.

Here, we focused our interest on the 12OAzo5AzoO12 dimer, a compound that was shown to exhibit chiral morphology and photoresponsivity originating from the presence of azobenzene moieties in molecular structure. We show that this material can host plasmonics Au NPs if these are grafted with proper ligands. Consequently, thin films with centimeter-scale ordered structures and chiral optical properties in both the organic and plasmonic spectral ranges were achieved. We also show that chiroptical properties of this system can be controlled with light and temperature. Large-area homochirality and ease of fabrication through assembly makes the proposed route a convenient alternative to advanced lithography methods,^45−47^ yielding large-scale 3D chiral systems with a wide range of self-organization dynamics control and dual-responsivity of the 3D structure.

## Results and Discussion

**Figure 1:**
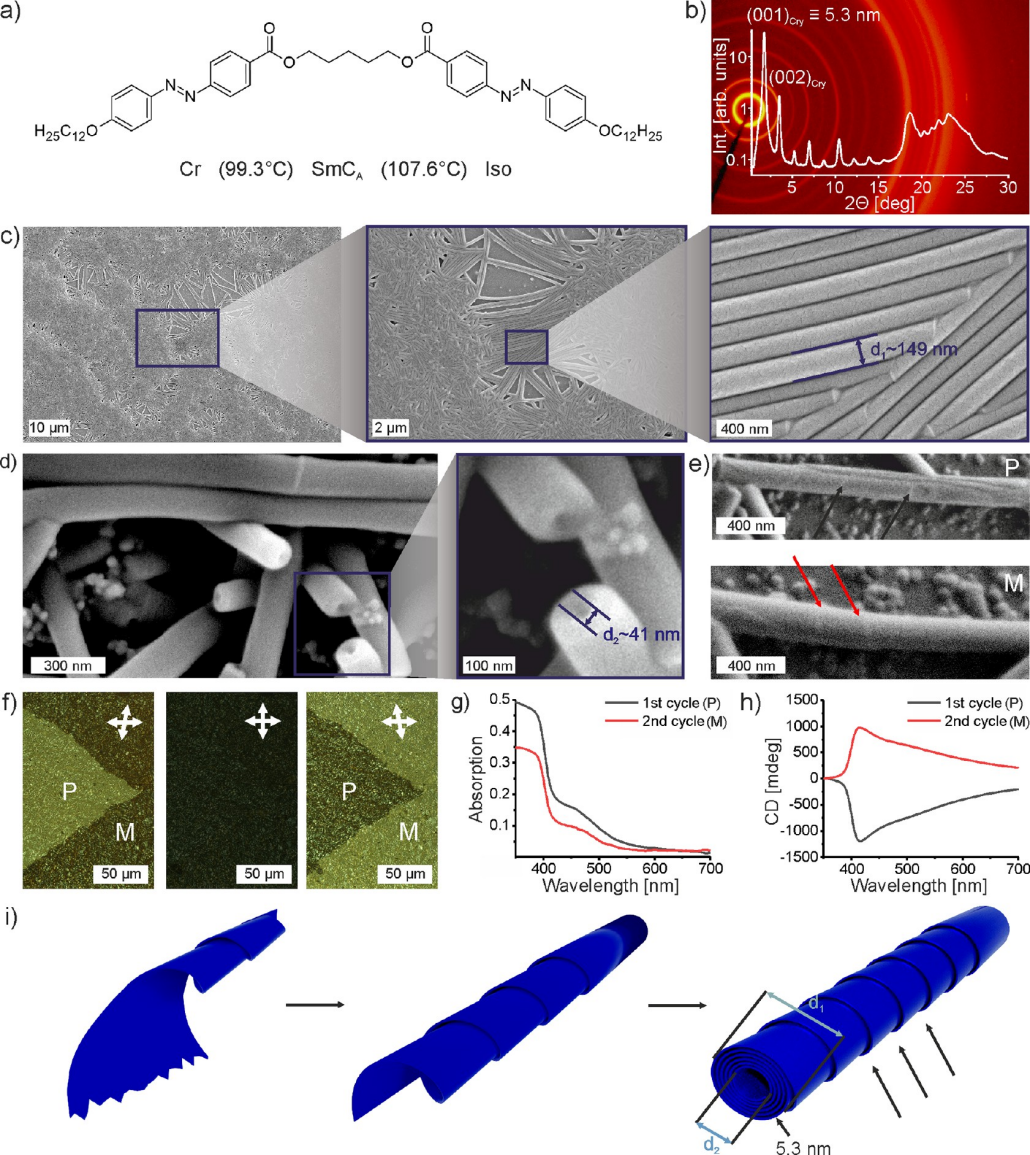
Structural and optical characterization of 12OAzo5AzoO12 dimer in thin films in crystal phase. a) Molecular formula and phase sequence. (b) X-ray diffractogram obtained at 30 °C (crystal phase). Main XRD peaks corresponding to interlayer distance in crystal phase are highlighted. (c–e) SEM images taken at different magnifications revealing (c) sample morphology, (d) internal voids of nanotubes, and (e) defects indicating that nanotubes are made from rolled layers; black and red arrows show defects for nanotubes of opposite handedness, *P* and *M*. (f) Polarizing optical microscopy images, directions of polarizers are indicated with arrows; slight decrossing of polarizers reveals formation of large optically active domains of the opposite sign. (g) UV–vis absorption spectra of samples after the first and second heat annealing cycle. (h) CD spectra corresponding to the absorption spectra shown in panel h. (i) 3D scheme of nanotube formation; *d*_1_ and *d*_2_ are inner and outer diameter of nanotube, respectively, corresponding to dimensions highlighted in panels (c) and (d).

The 12OAzo5AzoO12 compound (Figure 1a) was synthesized according to the previously reported synthetic path.^48^ Properties of the compound, which were previously reported to form helical nanoribbons, were re-examined. As will be discussed later, we detected different chiral morphology than that reported by Takezoe’s group, thus we carefully checked the purity of the synthesized compound to exclude the possibility that the difference is caused by small admixtures of impurities, e.g., coming from the decomposition of 12OAzo5AzoO12 dimer. ^1^H NMR and ^13^C NMR spectra did not reveal any signals indicating decomposition of 12OAzo5AzoO12 compound (Figure S1). The ^1^H NMR spectra enabled us to probe the geometry of N=N bonds. In a native sample, a system of four doublets was detected. In higher resolution, there is also an additional fine structure of doublets visible which is due to magnetic inequivalence of protons related to the para-substituted aromatic rings in the compound in *E*-isomer. After the UV irradiation, new signals were found: two signals between 6.7 and 6.9 ppm, and three signals overlapping with doublets from the *E* isomer, indicating coexistence of both *E* and *Z* isomers (Figure S2). We did not detect impurities by thin-layer chromatography (Figure S3a). Positive electrospray ionization time-of-flight mass spectrometry (TOF MS ES+) revealed two major peaks: at *m*/*z* 911 coming from a cluster of 12OAzo5AzoO12 with sodium cation [M – Na]^+^, and at 1802 coming from [2M + Na]^+^ (Figure S3b). Overall, the performed characterizations confirmed high purity and *E*- geometry of azo units for the 12OAzo5AzoO12 material in the native state. The following phase sequence for 12OAzo5AzoO12 compound on cooling: Iso (107.6 °C) SmC_A_ (93.7 °C) crystal was reported.^48^ Our studies (differential scanning calorimetry, DSC and X-ray diffraction, XRD) indicate that this compound melts directly to an isotropic liquid at 108.1 °C. On cooling, there are two phase transitions at 107.6 and 99.3 °C, corresponding to the formation of LC phase and crystal phase (Figure S4a–c), respectively, attesting a monotropic liquid crystalline behavior. The XRD diffractogram of the monotropic LC phase revealed smectic ordering, with short-range positional order inside the layers. The layer thickness is 2.8 nm, which corresponds to ca. half of the dimer length (Figure S 4d).

The XRD diffractogram of crystal phase (Figure 1b, Figure S4e), in low angle range, shows a number of sharp harmonic signals, characteristic for a lamellar crystal. The layer thickness in crystal phase corresponds to the full molecular length, 5.3 nm with small thermal expansion: −0.015 Å K^–1^. In the high angle range, XRD pattern proved that the layers are positionally correlated. Le and co-workers^49^ classified this phase as LC “Bx phase”; however based on our XRD studies, it should be considered as a lamellar type solid crystal. In line with XRD, polarizing optical microscopy (POM) observations with crossed polarizers revealed that below the isotropic phase, a schlieren texture with four and two brush defects is formed, which is characteristic to the anticlinic SmC phase^50^ (Figure S4f). On further cooling, at the transition to crystal phase, a texture with nearly zero birefringence is observed (Figure S4g). Decrossing polarizers by a few degrees revealed domains of different brightness that interchange upon changing direction of decrossing, which indicates optical activity of opposite signs in these crystal domains.

To probe the morphology of the crystal phase, we examined thin-film samples obtained by heat annealing of 12OAzo5AzoO12 material (Note S1). In scanning electron microscopy (SEM) micrographs, several microns large domains comprising bundles of parallel, close-packed, nanocylinders were visible (Figure 1c). Similar objects were previously described as heliconical nanotubes in the films of tris-biphenyl bent-core liquid crystals,^51,52^ chiral rod-like molecules,^53^ and acute-angle bent-core molecules based on naphthalene doped by nematogen.^54^ To determine factors playing the role in nanotubes formation, several tests were undertaken. Nanotubes obtained by solvent evaporation (Figure S5) were poorly ordered and short. Fast temperature quenching from the isotropic state, by immersing samples in liquid nitrogen, resulted in the formation of underdeveloped tubular structures (Figure S6). To produce well-developed nanotubes, the cooling rate 20 K min^–1^ or less has to be used. Careful studies of SEM images indicated that the nanotubes are hollow inside with inner diameter ∼41 nm (Figure 1d). The outer diameter of the nanotubes was dependent on cooling rate, average width was 147 ± 22, 139 ± 17 and 120 ± 19 nm for 20, 3, and 1 K min^–1^ cooling rate, respectively (Figure S7–S9). The external part of the tube bears features suggesting that it was formed by rolling of molecular layers (Figure 1e). Not surprisingly, given the achiral nature of 12OAzo5AzoO12 compound, tubules with left- and right-handed twist were found in samples. Interestingly, such nanotubular morphology for 12OAzo5AzoO12 compound has been noted also in previous studies by Takezoe et al., if material was dropcasted from mixtures with high temperature boiling solvents but only as a minor constituent in respect to the dominant helical nanoribbons.^49^ Potential source of differences between our results (no helical nanoribbons were observed) and those previously reported (helical nanoribbons were dominant) might be a different procedure for sample preparation or, which is less probable, a small degree of isomerization of azo bonds in the studied samples. It is known that twisted tubes and ribbons, although seem like very different morphologies,^51,52,55−57^ are actually closely related and can be tuned by Gaussian to mean curvature elastic energy balance.^58,59^ While the formation of twisted ribbons involves negative and positive local curvature of the membrane for the winded tubules, the positive (cylindrical) curvature is only required. The balance between positive and negative elastic curvature energy might depend on many factors like molecular shape as well as the crystallinity of the membrane.^60^ For the 12OAzo5AzoO12 compound studied here, a high tendency to form chiral assemblies of a nanotubular morphology by rolling of crystal layers is observed (Figure 1i). It is worth mentioning that seemingly nanotubular structures can be also formed by stacking of hollow cones;^61^ however, based on the TEM analysis, we were not able to find proof supporting this topology.

Given the above results, throughout the work, samples were prepared by dropcasting of 12OAzo5AzoO12 solution onto a glass plate, heating the sample to ∼130 °C (corresponding to the isotropic phase) and cooling to room temperature at 3 K min^–1^, if not stated otherwise (Note S1). In as prepared samples, POM revealed macroscopic (even centimeter large) domains with synchronized chirality (Figure 1f, Figure S10). This allowed us to perform solid state UV–vis and CD measurements at the single, homochiral domain (Figure 1g,h), without need to perform more demanding, microscopic UV–vis/CD measurements.^62^ For a thin film, absorption bands centered at 375 and 450 nm were detected, related to red-shifted π → π* and n → π* transitions of unsubstituted azobenzene,^63^ characteristic to molecules with *E* azobenzene configuration. CD measurements showed a strong asymmetric band with maximum at ∼410 nm and with opposite sign for domains with opposite optical activity. The dimensionless dissymmetry factor, g-factor, calculated at the CD band peak was ∼0.2, which is among the highest recorded for purely organic materials. The sign of CD signal correlates with sample morphology (Figure S11); based on SEM studies, we concluded that domains with the opposite CD signal are built of nanotubes of the opposite twist (Figure 1e,h), although, in principle, chirality could also originate from molecular order within the layers.^64,65^

### Photoswitchability of Organic Matrix

**Figure 2:**
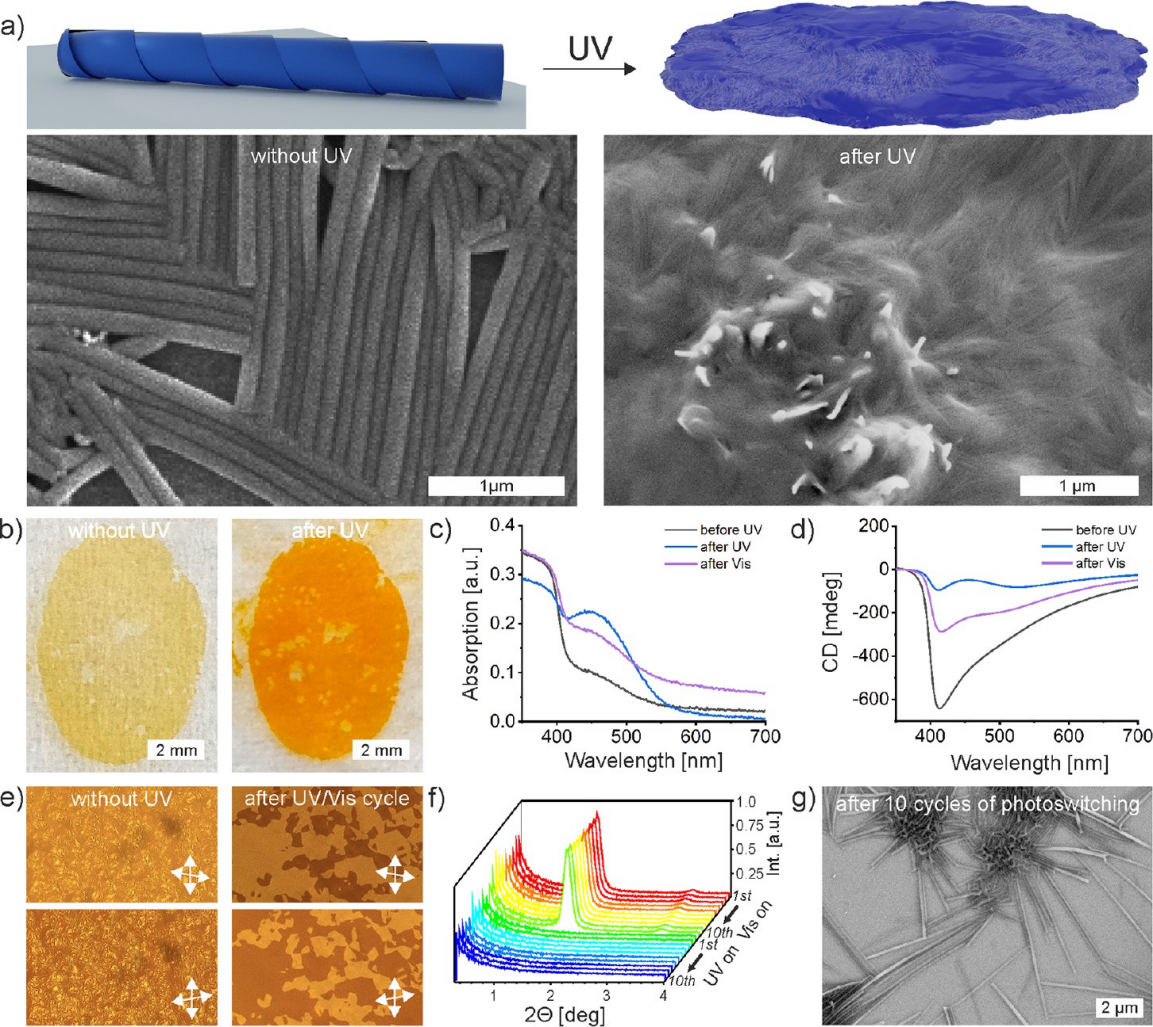
Photoswitching properties of 12OAzo5AzoO12 dimer. (a) SEM images revealing the change of sample morphology induced by UV irradiation and a model visualizing these changes. (b) Optical images of thin sample before and after UV radiation, sample irradiated with UV at 80 °C and cooled upon UV to room temperature has a noticeably different color. The sample area is about 0.5 cm^2^. (c) UV–vis absorption spectra at room temperature: native sample (before UV irradiation), subjected to UV irradiation (at 80 °C) and vis irradiation (at 80 °C). (d) CD spectra for samples showed in panel (c). (e) POM images (the presented area is ∼56 μm × 34 μm): Left: sample obtained by slow cooling, slight decrossing of polarizers reveals that the area is homochiral (up and bottom images show different direction of polarizers decrossing–domains having the opposite sign of optical activity); right: the same area irradiated by UV for 1 min at 80 °C and cooled down to room temperature (3 K min^–1^), upon UV irradiation small domains of opposite optical activity appeared. (f) Small angle X-ray diffraction for thin film recorded in sequence: UV on/vis on; the main diffraction peak corresponds to the thickness of layers in lamellar crystal. (g) SEM image of a sample exposed to 10 cycles of UV–vis switching.

We next probed structural and optical properties of the 12OAzo5AzoO12 compound when exposed to UV irradiation (Figure 2). A detailed description of the experiment is described in Note S2. Prepared samples were examined by XRD and SEM. In both cases, any structures other than amorphous aggregates were not observed (Figure 2a, Note S2, Figure S12a). For optical studies, a large, homochiral domain formed by the native, *E* isomer was prepared (Figure 2b, left). After irradiation, a distinct color change was noticed with the naked eye (Figure 2b, right). Accordingly, in UV–vis spectra of the sample, we noted a decrease in intensity absorption band centered at ∼375 nm, ascribed to π → π* transition (Figure 2c), what is characteristic for the *Z* isomer. Based on the relative intensity of this band before and after *E*/*Z* switching, following the equation proposed by Grossmann et al.,^63^ we can calculate that ca. 75% of azobenzene moieties adopted *Z* configuration (Note S2). This result is in agreement with CD measurements which revealed a CD band at ∼410 nm; however, the band intensity 10-fold decreased in comparison to the parent structure, suggesting that although some nanotubular structures are formed, the amorphous, achiral structure dominates (Figure 2d, Figure S12b,c). Switching dimer back to the *E* isomer was possible by irradiating sample with visible light for 30 s (Note S2), attested by the reappearance of absorption band centered at ∼375 nm in UV–vis spectra. Reappearance of the strong CD band at ∼410 nm confirmed restoring of tubular morphology (Figure 2c,d). POM revealed small chiral domains with small imbalance of chirality in the sample subject to vis irradiation (Figure 2e), explaining the lowered intensity of CD band in comparison to the parent sample. The reversibility of photoswitching was also followed with XRD and SEM. The dis- and reappearance of the lamellar XRD peak corresponding to 5.3 nm periodicity, characteristic to the *E* isomer, was monitored in at least 10 cycles of consecutive UV and Vis irradiation (Figure 2f). The formation of the nanotubes in sample exposed to 10 cycles of photoswitching was confirmed by SEM microscopy (Figure 2g).

Overall, in the context of fabricating chiroptical composites, the above results suggest that the 12OAzo5AzoO12 compound can be an excellent, responsive host for NPs: It readily melts and reforms nanotubes in UV and vis irradiation process, and it spontaneously forms macroscopic, homochiral domains. The nanotubes formed by rolling the crystal layers exhibit defects and internal voids, which, as previously shown for various nanotubular and LC materials,^66−71^ are able to accommodate dopants, including NPs.

### Nanoparticles

**Figure 3:**
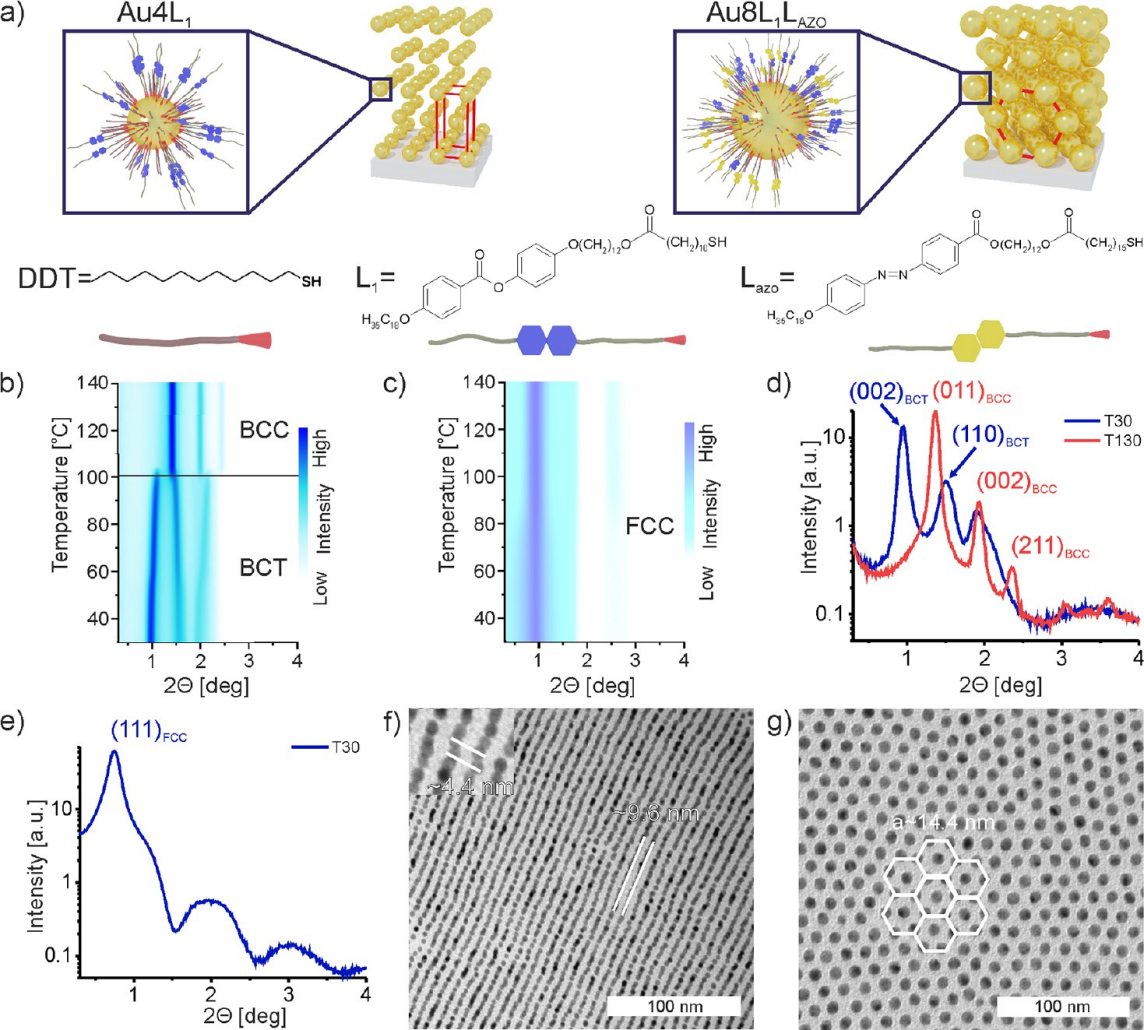
Structural investigation of Au NPs with diameter of ∼4.5 nm (Au4L_1_) and ∼8.5 nm (Au8L_1_L_AZO_) grafted with alkyl and LC-like ligands. (a) Schematic model of Au4L_1_ and Au8L_1_L_AZO_ Au NPs and molecular structure of ligands: DDT, L_1_, L_AZO_. (b, c) Temperature-dependent small angle XRD diffractograms of Au4L_1_ and Au8L_1_L_AZO_ materials (b, c, respectively) upon cooling. For the Au4L_1_ sample, a phase transition between BCC and BCT symmetries is highlighted. For Au8L_1_L_AZO_, FCC symmetry was detected in the measured temperature range. (d, e) 1D XRD diffractogram of Au4L_1_ sample at 30 and 130 °C and of Au8L_1_L_AZO_ material at 30 °C; XRD peaks characteristic to particular 3D symmetries of the samples are indexed. (f, g) TEM images of thermally annealed Au4L_1_ and Au8L_1_L_AZO_ (on the left and right, respectively) materials obtained by slow cooling (3 K min^–1^) to room temperature.

The goal of our studies was to obtain a hybrid material in which NPs are incorporated into an organic, chiral LC matrix, which requires that NPs are chemically compatible with the organic matrix enabling their good mixing in the isotropic phase and guiding NPs assembly when nanotubes are formed. Au NPs of 4.5 ± 0.4 nm diameter with dodecanethiol ligands were synthesized according to a well-established literature method^72^ (Note S3 and Figure S13a); to soften their organic shell and to achieve chemical compatibility to the organic host, a part of the dodecanethiols ligand was exchanged by a secondary, LC-like ligand (L_1_), yielding Au4L_1_ material according to the method described by Bagiński et al.^42^ (Figure 3a, Note S4). From XRD measurements (Figure 3b,d), we conclude that in thin-film form, Au4L_1_ NPs form two types of structures: body centered cubic (BCC) above 100 °C (Figure S13b) and body centered tetragonal (BCT) below 100 °C (Figure S13c). Apparently, NPs have deformable organic shell, which adopts toroidal shape at low temperatures (BCT phase), and spherical shape at elevated temperatures (BCC phase). The BCT structure was confirmed using TEM technique (Figure 3f) for samples cooled to room temperature. Namely, we identified vertically oriented layers of NPs with interlayer distance 9.6 nm, which corresponds to c/2 periodicity. Previously, we showed that such 2D TEM images represent the 3D BCT structure formed by NPs.

Since plasmonic properties of Au NPs strongly depend on their size, we synthesized also larger NPs^73^ with a diameter of 8.5 ± 0.5 nm and performed surface modification processes with the mesogenic ligand L_1_ (Notes S3 and S4, Figure S14). Unfortunately, the synthesized material showed only limited miscibility with the 12OAzo5AzoO12 compound (Figure S15); thus, to enhance the chemical compatibility, azobenzene ligand, L_AZO_, was additionally introduced into the grafting layer at the metal surface of NPs (Note S4, Figure S16). The best compatibility with matrix was achieved for NPs covered with equimolar ratio of L_1_ and L_AZO_ ligands - Au8L_1_L_AZO_ NPs (Figure 3a, right). As evidenced with XRD, Au8L_1_L_AZO_ NPs self-organized into face centered cubic (FCC) aggregates in the studied temperature range (Figure 3c,e). In line with XRD, heated to 140 °C and cooled down with 1 K min^–1^, the Au8L_1_L_AZO_ sample subject to TEM measurements revealed exclusively a hexagonal arrangement of NPs in a monolayer, with a center-to-center distance of 13.2–15.6 nm (Figure 3g, Figure S17a). In the tested temperature range, no effect of UV radiation on self-organization was observed (Figure S17b–f).

### Composite Materials

**Figure 4:**
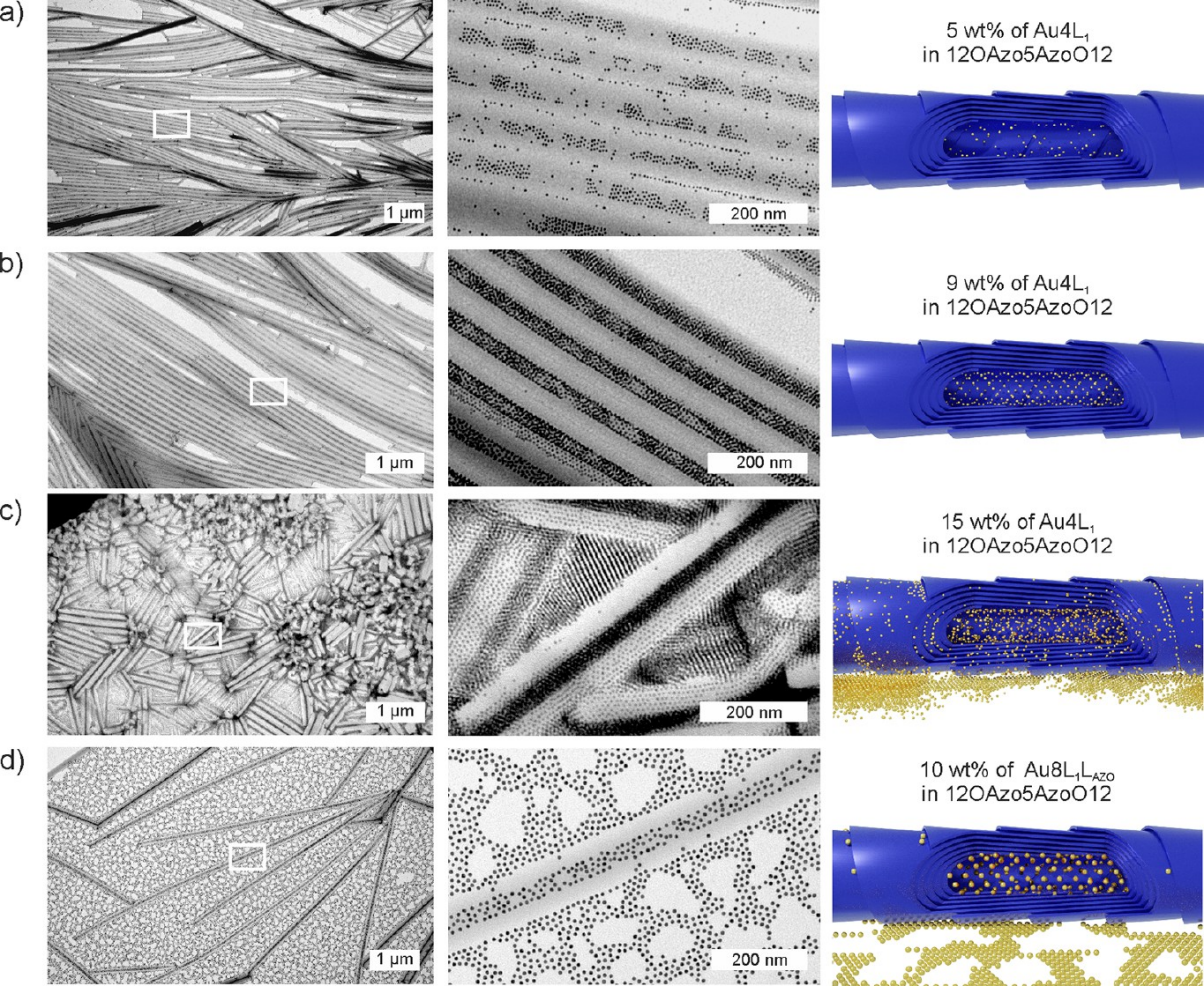
2D TEM characterization of 12OAzo5AzoO12/NP composite thin films after heat annealing. (a–c) Bright-field TEM images and schematic model of thermally annealed 12OAzo5AzoO12/Au4L_1_ composite with increasing amount of Au4L_1_ NPs (from a–c). White rectangles indicate a magnified area shown in the second column. (d) Schematic model and TEM images of thermally annealed 12OAzo5AzoO12/Au8L_1_L_AZO_ composite material.

Composites comprising the 12OAzo5AzoO12 dimer and different content of Au4L1 NPs were prepared (Note S5). The series of TEM and SEM experiments for mixtures with 5, 9, and 15 wt % Au NPs content are summarized in Figure 4a–c (Figure S18). In all cases, organic nanotubes with seemingly incorporated Au4L_1_ NPs were found (we analyze the 3D structure in detail below); however, the samples were not identical. Increasing the content of NPs, increased fill factor of nanotubes, although at the highest concentration, a large fraction of NPs forms also aggregates outside nanocylinders (Figure 4c). At 15 wt % and higher concentration of Au4L_1_ NPs, the process of folding organic layers into nanotubes becomes more difficult (Figure S18).

In samples with NP concentrations up to 9 wt %, the diameter of nanotubes is around 135 nm, slightly smaller than for pure organic nanotubes (Figure S18). For mixture with 9 wt % of Au4L_1_ NPs, we also tested the influence of cooling rate: 1, 3, and 20 K min^–1^ rates were used (Figure S19). The quickest cooling results in NPs were placed mainly at the edges of the tubes, which is probably a consequence of freezing of the system in the state of a local energy minimum, which in turn is associated with the limited time for organization of NPs in the organic matrix. At slower cooling rates, NPs are embedded mainly within the nanotubes centers. We thus decided to prepare all samples using a 3 K min^–1^ cooling rate to achieve well-ordered systems, while minimizing the time NPs remain at elevated temperature. This was crucial to prevent a thermally induced aggregation/reshaping of NPs. The change of organization of NPs with and without 12OAzo5AzoO12 was confirmed by XRD (Figure S20a). Additionally, temperature-dependent XRD measurements for 9 wt % of 12OAzo5AzoO12/Au4L_1_ were performed. This material is stable below 115 °C (Figure S20b,c).

In the case of Au8L_1_L_AZO_ NPs, several composites were prepared and tested (Figure S21); it was found that 10 wt % of NPs mixture is optimal to fully fill organic nanotubes (Figure 4d). The arrangement of NPs and the thermal stability of the optimal sample were investigated using X-ray methods (Figure S22).

**Figure 5:**
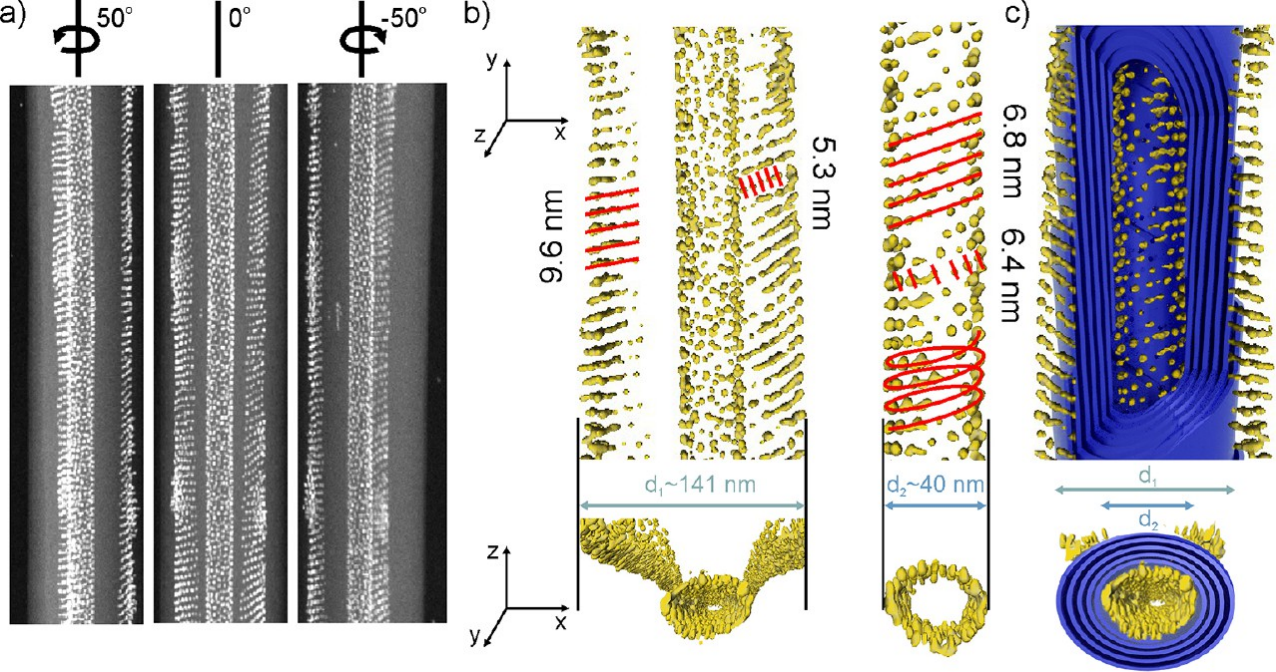
3D TEM characterization of 12OAzo5AzoO12/NP composite thin films after heat annealing. (a) Representative series of images acquired by tilting the sample, measurements performed in the HAADF-STEM mode. (b, c) 3D reconstruction of Au4L_1_ NPs organization based on measurements presented in panel (a); in panel (c), the reconstruction is overlaid onto the 12OAzo5AzoO12 nanocylinder model (blue). Characteristic dimensions of nanotubes and interparticle distances are highlighted with bluish and red lines, respectively.

In order to unequivocally determine the 3D arrangement of the NPs inside the nanocylinders, a sample with 9 wt % of Au4L_1_ NPs in 12OAzo5AzoO12 matrix was further studied using a TEM tomographic method in the high-angle annular dark-field scanning transmission electron microscopy (HAADF-STEM) mode (Supplementary Movies 1 and 2). The series of images was acquired by tilting the sample between −56° and +60° with 2° step, representative HAADF-STEM projection images of the series are presented in Figure 5a. These images revealed weakly scattered organic nanotubes in contact with two types of NPs assemblies, placed outside and inside the organic nanotubes. NPs outside the nanotubes are built with regularly spaced rows of NPs with inter- and in-row distances of ∼9.6 nm and ∼5.3 nm, respectively (Figure S23a,b); normal to the rows is at an oblique angle to the nanotube main axis. The distance ∼9.6 nm is similar to the c/2 dimension of the BCT unit cell identified in films of Au4L_1_ NPs, attesting a large role of L_1_ ligands in the assembly of this fraction of NPs. In contrast, NPs inside the nanotubes are helically organized, with a helix diameter of ∼40 nm, corresponding to the estimated internal diameter of an organic nanotube; the axis of helix is colinear with the axis of nanotubes (Figure 5b), the helical pitch is ∼6.8 nm, whereas the distance of NPs along the helix is ∼6.4 nm, suggesting a rather isotropic distribution of ligands around the NP core, as in case of BCC structure identified for purely NPs films at an elevated temperature (Figure S23c,d). It also worth noting that the helical pitch of helix formed by NPs is close to the curvature of organic layers forming the organic nanotubes, thus we can assume that NP strongly interacts with the inner surface of nanotubes. The different assembly mode of NPs inside and outside nanotubes reflects the soft, deformable character of the organic shell of NPs and highlights the benefits of using LC-like ligands. To fully appreciate the relation between NPs organization and structure of organic nanotubes, the tomographic reconstruction of NPs assemblies was overlaid onto a 3D, sliced model of organic nanotubes (Figure 5c). 3D reconstruction of 12OAzo5AzoO12/Au8L_1_L_AZO_ material revealed the presence of NPs at the surface and within the void of organic nanotubes (Figures S24 and S25, Supplementary Movies 3 and 4).

**Figure 6:**
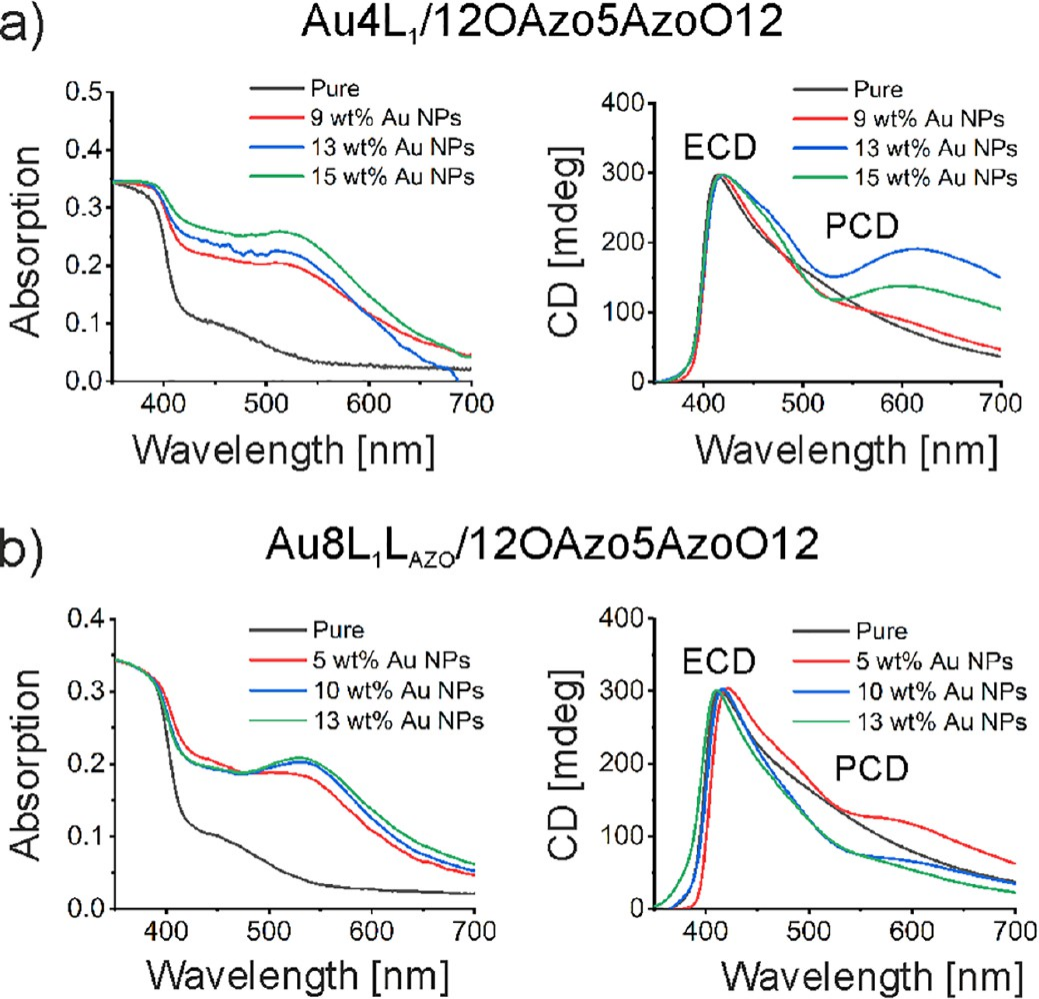
Optical characterization of 12OAzo5AzoO12/NP composite thin films with varied amounts of NPs: UV–vis absorption spectra and CD spectroscopy for composites with Au4L_1_ NPs (a) and Au8L_1_L_AZO_ (b). Regions in which electronic and plasmonic CD bands appear are highlighted as ECD and PCD.

After careful examination of the composite structures, we tested the optical properties of materials. UV–vis measurements revealed that these materials exhibit absorption bands characteristic to the 12OAzo5AzoO12 dimer at 375 nm and plasmonic absorption of NPs above 500 nm (Figure 6).

It is worth noting that plasmonics absorption bands are relatively broad and shifted toward longer wavelengths, in comparison to those observed for NP dispersions in solution (Figures S26 and S27). These features indicate plasmonic coupling between NPs, which is not surprising given the relatively small particle-to-particle distance measured with XRD. Notably, plasmonics coupling of helically arranged NPs gives a chiral optical response from achiral NPs.^70^ CD measurements of homochiral domains of composites revealed not only CD bands characteristic to the organic material (centered at ∼410 nm) but also an additional signal centered at ∼650 nm, close to the detected plasmonic absorption. The wavelength of the noted CD band suggests it is a positive part of a Cotton band; the full Cotton characteristic is not clearly observed due to the close proximity of a strong CD band of organic 12OAzo5AzoO12 material.

**Figure 7:**
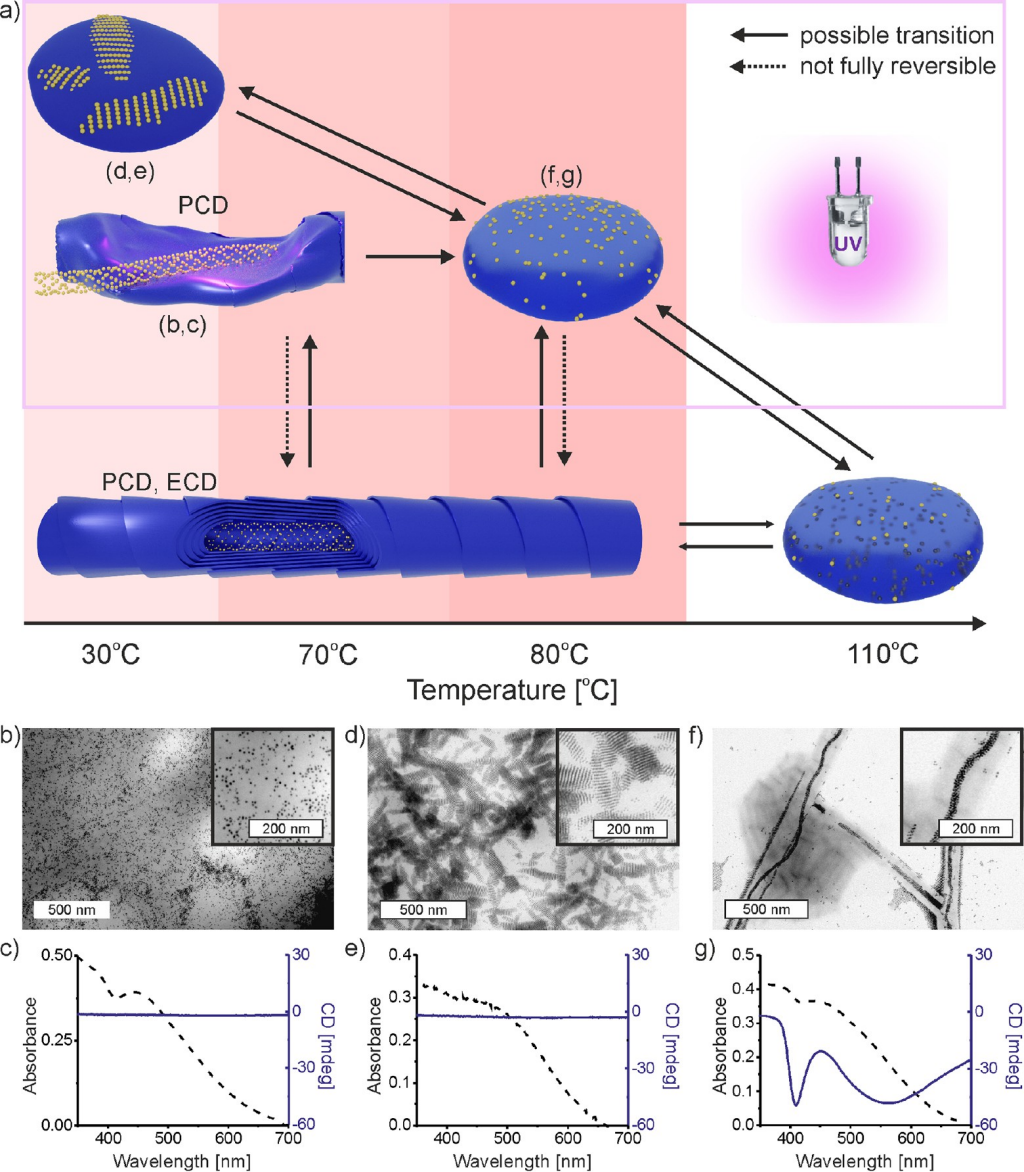
Multistate control of 12OAzo5AzoO12/NP composite thin films by thermal and UV stimuli. (a) Schematic diagram showing varied geometries of composite depending on UV/thermal treatment of the sample; varied chiral properties are indicated (ECD, PCD); some transitions are marked as not fully reversible, as the increase of matrix polarity upon UV exposure results in the loss of composite homogeneity. (b, d, f) Representative TEM images. (c, e, g) UV–vis and CD spectra of samples.

Finally, multiresponsivity of the composites was checked, i.e., switching between different states by means of temperature and/or light. Systematic studies of the influence of these two stimuli allowed us to build a diagram of the 12OAzo5AzoO12/Au4L_1_ composite behavior (Figure 7a, Note S6). At elevated temperature (∼115 °C), the sample melts and becomes amorphous, it does not exhibit chiroptical properties, neither electronic circular dichroism (ECD) nor plasmonics circular dichroism (PCD) was found. In this case, NPs are well dispersed within the volume of the organic host. Lowering the temperature leads to cocrystallization of 12OAzo5AzoO12 and NPs, and as a result, PCD and ECD signals reappear. The melting/crystallization-based responsiveness does not differ much from what was detected for purely organic films. However, the diagram becomes more complex when considering the effect of UV light irradiation as the second stimuli. Studies on the combined effects of temperature and UV light allowed us to identify three distinct structural states, which differ in the spatial distribution of NPs in the 12OAzo5AzoO12 matrix. TEM images revealed that UV irradiation at 80 °C–100 °C, followed by an abrupt lowering of temperature “freezes”, an amorphous state of dimer aggregates (in Z-configuration) and results in a random distribution of NPs that is mainly located at the surface of the organic material (Figure 7b). Slow lowering of temperature (3 K min^–1^) from 80 °C upon UV on led to the assembly of NPs into layers (Figure 7d). In this case, NPs most probably form the BCT structure at the surface of the organic material, which is attested by the ∼8.3 nm interlayer distance similar to the c/2 dimension of the BCT unit cell. In both cases, UV-induced *E* to *Z* isomerization of azo moiety translates to an increase of the matrix polarity. This apparently leads to phase separation of NPs from the organic material, where NPs are pushed out from the polar matrix. In both described cases, the absorption band of 12OAzo5AzoO12 and plasmonic band of NPs are merged into a single, broad signal placed at 450–500 nm. These bands are not accompanied by a CD response (Figure 7c,e, Figures S28–S30).

An intriguing structural state was obtained for UV irradiation at 70 °C, followed by abrupt lowering of temperature. TEM images showed that in most areas, organic nanotubes are melted, while NPs preserve a nanotubular structure (Figure 7f) characteristic to NPs assemblies formed within organic nanotubes of a nonirradiated sample. Indeed, these NPs tubules are found within an amorphous organic material, while their diameter is ∼32 nm, suggesting they were previously within the centers of organic nanotubes (Figure S31).

Although these assemblies are deformed in comparison to the parent structure analyzed in Figures 4–6, we decided to test if NPs preserve helical ordering that is exhibited in chiroptical properties. CD spectroscopy revealed a significant reduction of intensity of the CD band at 410 nm, which is characteristic to organic nanotubes, while plasmonic the CD band remained strong (Figure 7g, Figure S32). We suggest that NPs interact strongly (note that 100 °C is required to melt NPs in the neat form), while an increase in organic polarity (adopting the *Z* configuration) efficiently decreases NP/dimer interactions; both these effects cause tubular structures of NPs to endure the melting of the organic “template” that was used to form the assembly.

## Conclusions

In conclusion, we developed a strategy for fabricating chiral assemblies of achiral NPs, using an organic liquid crystal template. The organic material, built of achiral mesogenic dimers, spontaneously forms supramolecular, hollow, helical nanotubes upon the phase transition to the crystal phase. These nanotubes serve as “nanocapillaries” which can be filled with NPs and generate a helical assembly of NPs. Formation of such structures is an excellent example of spontaneous symmetry breaking that induces symmetry breaking of another (doped) material. Due to the presence of the azo group in molecular structures of organic matrices, both nanotubes and helices of NPs can be melted and restored by UV and vis light absorption, without the presence of solvent, overcoming an important limitation for many applications requiring thin-film forms of chiroptical materials. These composite materials show centimeter-scale domains exhibiting strong circular dichroism related to plasmonic and organic excitations. The presented approach affords capabilities of remotely controlled chirality with multistate structural control of organic and NP components, further advancing the development of stimuli-responsive chiroptical assembled systems, which is particularly interesting in the view of structures presented by Gansel et al.^74^ on the chirality-based metamaterial and transistor technologies with nonlithographically organized active parts.^75^

## Experimental Section

### Chemicals

All chemicals were used as purchased, without any further purification: tetrachloroauric (III) acid trihydrate (Sigma-Aldrich, ≥99.9% trace metals basis), formaldehyde solution 37–41% (Fischer Chemical, analytical reagent grade, stabilized with ca. 12% methanol), dodecylamine (Acros Organics, 98%), dodecanethiol (Sigma-Aldrich, ≥98%), oleylamine (TCI, >50%), 1,2,3,4-tetrahydronaphthalene (Fisher Chemicals, ≥97%), tetrabutylammonium bromide (Sigma-Aldrich, ACS reagent, ≥98.0%). All reagents for organic synthesis were obtained from Sigma-Aldrich. The reaction products were purified by column chromatography using SiliCycle Silia Flash P60 (40–63 μm, 60 Å) at an atmospheric pressure or by crystallization. Thin-layer chromatography was performed using a silica gel 60 Å F254 (Merck) precoated aluminum substrate and visualized using iodine vapor and/or a UV lamp (254 nm). All solvents were obtained from Sigma-Aldrich.

### NMR Measurements

^1^H NMR and ^13^C NMR spectra were recorded using a 500 MHz NMR Varian Unity Plus in CDCl_3_. Proton chemical shifts were reported in ppm (δ) relative to the internal standard – tetramethylsilane (δ= 0.00 ppm). Carbon chemical shifts are reported in ppm (δ) relative to the residual solvent signal (CDCl_3_, δ = 77.0 ppm).

### XRD Measurements

XRD measurements at small angles were performed with a Bruker Nanostar system (Cu K α radiation, parallel beam formed by cross-coupled Goebel mirrors, and a 3-pinhole collimation system, VANTEC 2000 area z detector). The temperature of the sample was controlled with a precision of 0.1 K. Samples were prepared as thin films on Kapton tape or silica wafer substrates. X-ray diffractograms at wide angles were obtained with the Bruker D8 GADDS system (Cu Kα line, Goebel mirror, point beam collimator, Vantec2000 area detector). Experimental diffractograms were analyzed using Topas 3 software (Bruker). Samples were prepared as thin films on Kapton tape or silica wafer substrates.

### Transmission Electron Microscopy

TEM measurements were performed using the following equipment: a high-resolution JEM 1400 microscope (JEOL Co., Japan) equipped with tomographic holder and high-resolution digital camera CCD MORADA G2 (EMSIS GmbH, Germany) at Nencki Institute of Experimental Biology of Polish Academy of Sciences and a model JEM – 1011 (JEOL) transmission electron microscope equipped with a model EDS INCA (Oxford) analyzer (Mossakowski Medical Research Centre Polish Academy of Sciences, Warsaw).

### Transmission Electron Tomography

TEM investigations were conducted using a Thermo Scientific Talos F200X transmission microscope at 200 kV. The measurements were performed in STEM mode using the high-angle annular dark-field (HAADF) detector. Thermo Scientific Tomography software was used for the acquisition of individual HAADF images. Inspect 3D ver. 4.4 and Amira Life Sciences 6.2.0 software were used to obtain 3D reconstructions. Three algorithms such as weighted back-projection, simultaneous iterative reconstructive technique, and expectation–maximization (EM) were tested to reconstruct the structure. Finally, the 3D structure was obtained using an EM algorithm with 30 iterations (criterion: the best brightness–contrast of the final image).

### Scanning Electron Microscopy

SEM investigation was conducted using the FE-SEM/EDS, available at the Faculty of Chemistry, University of Warsaw and ZEISS SIGMA VP scanning electron microscope at the Faculty of Geology, University of Warsaw. The imaging was realized on Si wafers or glass plates. The samples were sputtered with a 5 nm gold layer to improve contrast.

### Differential Scanning Calorimetry

Calorimetric studies were performed with the TA DSC Q200 microcalorimeter. The sample with a mass of 3 mg was sealed in aluminum pans and kept in nitrogen atmosphere during the measurement; both heating and cooling scans with a rate of 5 K min^–1^ were applied.

### Polarized Optical Microscopy

POM observations were carried out under the Zeiss Imager A2m polarizing microscope equipped with the Linkam heating stage. Samples were observed in glass cells with various thickness of 1.5 to 10 μm or on glass substrates.

### UV–vis Measurements

Spectroscopic study of the materials in the colloid in the UV–vis range were performed using GENESYS 50 UV–vis spectrometer. The spectra of the functionalized NPs were performed in THF solutions, using quartz cuvettes with a 1 mm optical path.

### Circular Dichroism Measurements

CD investigations were recorded using Chirascan Circular Dichroism Spectrometer by Applied PhotoPhysics. The data are not corrected for reflection.

### NPs Synthesis

Two types of Au NPs were synthesized: spherical Au NPs with an average diameter of 4.5 nm (Au4@DT) and spherical Au NPs with an average diameter 8.5 nm. Syntheses were conducted according to the literature methods^72,73^ (Note 3). LC-like ligand L_1_ and azobenzene ligand L_AZO_ were introduced to the NP surface using literature methods^35,42,76^ (Note 4).

### Preparation of Hybrid Nanomaterial

The following description exemplified the preparation of the 12OAzo5AzoO12/Au4L_1_ composite material when using the optimal parameters (cooling rate and component ratio) and a TEM grid as a substrate. The remaining samples were prepared in an analogous manner. A total of 6 μL of 0.5 mg mL^–1^ dispersion of Au4L_1_ NPs in toluene was mixed with 20 μL of 2 mg mL^–1^ solution of 12OAzo5AzoO12 in THF. Then, the mixture was sonicated, and 3 μL of the mixture was dropcast onto a TEM grid. Next, the TEM grid was placed onto a heating table and subject to heating/cooling cycle between 30 and 130 °C, with a cooling rate of 3 °C min^–1^ and heating rate of 20 °C min^–1^.

## Supporting Information Available

Confirmation of purity and liquid-crystalline properties of 12OAzo5AzoO12 compound, additional structural investigation of pure organic material, structural characterization of Au4 and Au8 NPs, synthetic procedures of L_AZO_ compound, additional structural and optical analysis of composite materials (PDF)
Movie S1: Supplementary Movies are grayscale and colored visualizations of the HAADF-STEM tomography corresponding to NP/12OAzo5AzoO12 composite thin films after heat annealing: Au4L/12OAzo5AzoO12 (MP4)
Movie S2: Supplementary Movies are grayscale and colored visualizations of the HAADF-STEM tomography corresponding to NP/12OAzo5AzoO12 composite thin films after heat annealing: Au4L/12OAzo5AzoO12 (MP4)
Movie S3: Au8L_1_L_AZO_/12OAzo5AzoO12 (MP4)
Movie S4: Au8L_1_L_AZO_/12OAzo5AzoO12 (MP4)

## References

[ref1] Yu N.; Huang L.; Zhou Y.; Xue T.; Chen Z.; Han G. Near-Infrared-Light Activatable Nanoparticles for Deep-Tissue-Penetrating Wireless Optogenetics. Adv. Healthc. Mater. 2019, 8 (6), 1801132 10.1002/adhm.201801132. 30633858

[ref2] Pastoriza-Santos I.; Kinnear C.; Pérez-Juste J.; Mulvaney P.; Liz-Marzán L. M. Plasmonic Polymer Nanocomposites. Nat. Rev. Mater. 2018, 3 (10), 375–391. 10.1038/s41578-018-0050-7.

[ref3] Siampour H.; Kumar S.; Davydov V. A.; Kulikova L. F.; Agafonov V. N.; Bozhevolnyi S. I. On-Chip Excitation of Single Germanium Vacancies in Nanodiamonds Embedded in Plasmonic Waveguides. Light Sci. Appl. 2018, 7, 61 10.1038/s41377-018-0062-5. 30245809 PMC6134053

[ref4] Stoychev G.; Kirillova A.; Ionov L. Light-Responsive Shape-Changing Polymers. Adv. Opt. Mater. 2019, 7, 1900067 10.1002/adom.201900067.

[ref5] Albano G.; Pescitelli G.; Di Bari L. Chiroptical Properties in Thin Films of π-Conjugated Systems. Chem. Rev. 2020, 120 (18), 10145–10243. 10.1021/acs.chemrev.0c00195. 32892619

[ref6] Kuzyk A.; Yang Y.; Duan X.; Stoll S.; Govorov A. O.; Sugiyama H.; Endo M.; Liu N. A Light-Driven Three-Dimensional Plasmonic Nanosystem That Translates Molecular Motion into Reversible Chiroptical Function. Nat. Commun. 2016, 7, 10591 10.1038/ncomms10591. 26830310 PMC4740900

[ref7] Willner E. M.; Kamada Y.; Suzuki Y.; Emura T.; Hidaka K.; Dietz H.; Sugiyama H.; Endo M. Single-Molecule Observation of the Photoregulated Conformational Dynamics of DNA Origami Nanoscissors. Angew. Chemie - Int. Ed. 2017, 56 (48), 15324–15328. 10.1002/anie.201708722. 29044955

[ref8] Du M.; Ribeiro R. F.; Yuen-Zhou J. Remote Control of Chemistry in Optical Cavities. Chem. 2019, 5 (5), 1167–1181. 10.1016/j.chempr.2019.02.009.

[ref9] Shalaev M. I.; Walasik W.; Litchinitser N. M. Optically Tunable Topological Photonic Crystal. Optica 2019, 6 (7), 839 10.1364/OPTICA.6.000839.

[ref10] Pang X.; Lv J. an; Zhu C.; Qin L.; Yu Y. Photodeformable Azobenzene-Containing Liquid Crystal Polymers and Soft Actuators. Adv. Mater. 2019, 31 (52), 1904224 10.1002/adma.201904224. 31595576

[ref11] Chen H.; Hou A.; Zheng C.; Tang J.; Xie K.; Gao A. Light- and Humidity-Responsive Chiral Nematic Photonic Crystal Films Based on Cellulose Nanocrystals. ACS Appl. Mater. Interfaces 2020, 12 (21), 24505–24511. 10.1021/acsami.0c05139. 32362108

[ref12] Zhang X.; Xu Y.; Valenzuela C.; Zhang X.; Wang L.; Feng W.; Li Q. Liquid Crystal-Templated Chiral Nanomaterials : From Chiral Plasmonics to Circularly Polarized Luminescence. Light Sci. Appl. 2022, 11, 223 10.1038/s41377-022-00913-6. 35835737 PMC9283403

[ref13] Oh S. W.; Nam S. M.; Kim S. H.; Yoon T. H.; Kim W. S. Self-Regulation of Infrared Using a Liquid Crystal Mixture Doped with Push-Pull Azobenzene for Energy-Saving Smart Windows. ACS Appl. Mater. Interfaces 2021, 13 (4), 5028–5033. 10.1021/acsami.0c19015. 33472366

[ref14] Kumar K.; Knie C.; Bléger D.; Peletier M. A.; Friedrich H.; Hecht S.; Broer D. J.; Debije M. G.; Schenning A. P. H. J. A Chaotic Self-Oscillating Sunlight-Driven Polymer Actuator. Nat. Commun. 2016, 7, 11975 10.1038/ncomms11975. 27375235 PMC4932179

[ref15] Hrozhyk U. A.; Serak S. V.; Tabiryan N. V.; Bunning T. J. Optical Tuning of the Reflection of Cholesterics Doped with Azobenzene Liquid Crystals. Adv. Funct. Mater. 2007, 17 (11), 1735–1742. 10.1002/adfm.200600776.

[ref16] Zhang L.; Pan J.; Liu Y.; Xu Y.; Zhang A. NIR-UV Responsive Actuator with Graphene Oxide/Microchannel-Induced Liquid Crystal Bilayer Structure for Biomimetic Devices. ACS Appl. Mater. Interfaces 2020, 12 (5), 6727–6735. 10.1021/acsami.9b20672. 31917536

[ref17] Poryvai A.; Smahel M.; Svecova M.; Nemati A.; Shadpour S.; Ulbrich P.; Ogolla T.; Liu J.; Novotna V.; Veverka M.; Vejpravova J.; Hegmann T.; Kohout M. Chiral, Magnetic, and Photosensitive Liquid Crystalline Nanocomposites Based on Multifunctional Nanoparticles and Achiral Liquid Crystals. ACS Nano 2022, 16 (8), 11833–11841. 10.1021/acsnano.1c10594. 35867644

[ref18] Choi S. W.; Izumi T.; Hoshino Y.; Takanishi Y.; Ishikawa K.; Watanabe J.; Takezoe H. Circular-Polarization-Induced Enantiomeric Excess in Liquid Crystals of an Achiral, Bent-Shaped Mesogen. Angew. Chemie - Int. Ed. 2006, 45 (9), 1382–1385. 10.1002/anie.200503767. 16440382

[ref19] Ueda T.; Masuko S.; Araoka F.; Ishikawa K.; Takezoe H. A General Method for the Enantioselective Formation of Helical Nanofilaments. Angew. Chemie - Int. Ed. 2013, 52 (27), 6863–6866. 10.1002/anie.201300658. 23716417

[ref20] Park W.; Ha T.; Kim T. T.; Zep A.; Ahn H.; Shin T. J.; Sim K. I.; Jung T. S.; Kim J. H.; Pociecha D.; Gorecka E.; Yoon D. K. Directed Self-Assembly of a Helical Nanofilament Liquid Crystal Phase for Use as Structural Color Reflectors. NPG Asia Mater. 2019, 11, 45 10.1038/s41427-019-0146-6.

[ref21] Park W.; Wolska J. M.; Pociecha D.; Gorecka E.; Yoon D. K. Direct Visualization of Optical Activity in Chiral Substances Using a Helical Nanofilament (B4) Liquid Crystal Phase. Adv. Opt. Mater. 2019, 7 (23), 1901399 10.1002/adom.201901399.

[ref22] Zhang Q.; Hernandez T.; Smith K. W.; Jebeli S. A. H.; Dai A. X.; Warning L.; Baiyasi R.; McCarthy L. A.; Guo H.; Chen D. H.; Dionne J. A.; Landes C. F.; Link S. Unraveling the Origin of Chirality from Plasmonic Nanoparticle-Protein Complexes. Science 2019, 365 (6460), 1475–1478. 10.1126/science.aax5415. 31604278

[ref23] Zhou M.; Sang Y.; Jin X.; Chen S.; Guo J.; Duan P.; Liu M. Steering Nanohelix and Upconverted Circularly Polarized Luminescence by Using Completely Achiral Components. ACS Nano 2021, 15 (2), 2753–2761. 10.1021/acsnano.0c08539. 33559470

[ref24] Zhao B.; Gao X.; Pan K.; Deng J. Chiral Helical Polymer/Perovskite Hybrid Nanofibers with Intense Circularly Polarized Luminescence. ACS Nano 2021, 15 (4), 7463–7471. 10.1021/acsnano.1c00864. 33724002

[ref25] Shi Y.; Duan P.; Huo S.; Li Y.; Liu M. Endowing Perovskite Nanocrystals with Circularly Polarized Luminescence. Adv. Mater. 2018, 30 (12), 1705011 10.1002/adma.201705011. 29363205

[ref26] King M. E.; Fonseca Guzman M. V.; Ross M. B. Material Strategies for Function Enhancement in Plasmonic Architectures. Nanoscale 2022, 14 (3), 602–611. 10.1039/D1NR06049J. 34985484

[ref27] Rao A.; Roy S.; Jain V.; Pillai P. P. Nanoparticle Self-Assembly: From Design Principles to Complex Matter to Functional Materials. ACS Appl. Mater. Interfaces 2022. 10.1021/acsami.2c05378 35715224

[ref28] Wu W.; Pauly M. Chiral Plasmonic Nanostructures: Recent Advances in Their Synthesis and Applications. Mater. Adv. 2022, 3 (1), 186–215. 10.1039/D1MA00915J.

[ref29] Kong X. T.; Besteiro L. V.; Wang Z.; Govorov A. O. Plasmonic Chirality and Circular Dichroism in Bioassembled and Nonbiological Systems: Theoretical Background and Recent Progress. Adv. Mater. 2020, 32 (41), 1801790 10.1002/adma.201801790. 30260543

[ref30] Chen C. L.; Zhang P.; Rosi N. L. A New Peptide-Based Method for the Design and Synthesis of Nanoparticle Superstructures: Construction of Highly Ordered Gold Nanoparticle Double Helices. J. Am. Chem. Soc. 2008, 130 (41), 13555–13557. 10.1021/ja805683r. 18800838 PMC5765746

[ref31] Neubrech F.; Hentschel M.; Liu N. Reconfigurable Plasmonic Chirality: Fundamentals and Applications. Adv. Mater. 2020, 32 (41), 1905640 10.1002/adma.201905640. 32077543

[ref32] Pawlak M.; Bagiński M.; Llombart P.; Beutel D.; González-Rubio G.; Górecka E.; Rockstuhl C.; Mieczkowski J.; Pociecha D.; Lewandowski W. Tuneable Helices of Plasmonic Nanoparticles Using Liquid Crystal Templates: Molecular Dynamics Investigation of an Unusual Odd-Even Effect in Liquid Crystalline Dimers. Chem. Commun. 2022, 58 (53), 7364–7367. 10.1039/D2CC00560C. 35621065

[ref33] Zhou C.; Duan X.; Liu N. A Plasmonic Nanorod That Walks on DNA Origami. Nat. Commun. 2015, 6, 8102 10.1038/ncomms9102. 26303016 PMC4560816

[ref34] Ryssy J.; Natarajan A. K.; Wang J.; Lehtonen A. J.; Nguyen M. K.; Klajn R.; Kuzyk A. Light-Responsive Dynamic DNA-Origami-Based Plasmonic Assemblies. Angew. Chemie - Int. Ed. 2021, 60 (11), 5859–5863. 10.1002/anie.202014963. PMC7986157 33320988

[ref35] Bagiński M.; Tupikowska M.; González-Rubio G.; Wójcik M.; Lewandowski W. Shaping Liquid Crystals with Gold Nanoparticles: Helical Assemblies with Tunable and Hierarchical Structures Via Thin-Film Cooperative Interactions. Adv. Mater. 2020, 32 (1), 1904581 10.1002/adma.201904581. 31729083

[ref36] Grzelak D.; Tupikowska M.; Vila-Liarte D.; Beutel D.; Bagiński M.; Parzyszek S.; Góra M.; Rockstuhl C.; Liz-Marzán L. M.; Lewandowski W. Liquid Crystal Templated Chiral Plasmonic Films with Dynamic Tunability and Moldability. Adv. Funct. Mater. 2022, 32, 2111280 10.1002/adfm.202111280.

[ref37] Wojcik M.; Kolpaczynska M.; Pociecha D.; Mieczkowski J.; Gorecka E. Multidimensional Structures Made by Gold Nanoparticles with Shape-Adaptive Grafting Layers. Soft Matter 2010, 6 (21), 5397–5400. 10.1039/c0sm00539h.

[ref38] Wojcik M.; Lewandowski W.; Matraszek J.; Mieczkowski J.; Borysiuk J.; Pociecha D.; Gorecka E. Liquid-Crystalline Phases Made of Gold Nanoparticles. Angew. Chemie - Int. Ed. 2009, 48 (28), 5167–5169. 10.1002/anie.200901206. 19496089

[ref39] Parzyszek S.; Pociecha D.; Wolska J. M.; Lewandowski W. Thermomechanically Controlled Fluorescence Anisotropy in Thin Films of InP/ZnS Quantum Dots. Nanoscale Adv. 2021, 3 (18), 5387–5392. 10.1039/D1NA00290B. 36132630 PMC9418115

[ref40] Grzelak D.; Parzyszek S.; Moroz P.; Szustakiewicz P.; Zamkov M.; Lewandowski W. Self-Assembled PbS/CdS Quantum Dot Films with Switchable Symmetry and Emission. Chem. Mater. 2019, 31 (19), 7855–7863. 10.1021/acs.chemmater.9b01767.

[ref41] Lewandowski W.; Fruhnert M.; Mieczkowski J.; Rockstuhl C.; Górecka E. Dynamically Self-Assembled Silver Nanoparticles as a Thermally Tunable Metamaterial. Nat. Commun. 2015, 6, 6590 10.1038/ncomms7590. 25779822

[ref42] Bagiński M.; Pedrazo-Tardajos A.; Altantzis T.; Tupikowska M.; Vetter A.; Tomczyk E.; Suryadharma R. N. S.; Pawlak M.; Andruszkiewicz A.; Górecka E.; Pociecha D.; Rockstuhl C.; Bals S.; Lewandowski W. Understanding and Controlling the Crystallization Process in Reconfigurable Plasmonic Superlattices. ACS Nano 2021, 15 (3), 4916–4926. 10.1021/acsnano.0c09746. 33621046 PMC8028333

[ref43] Tomczyk E.; Promiński A.; Bagiński M.; Górecka E.; Wójcik M. Gold Nanoparticles Thin Films with Thermo- and Photoresponsive Plasmonic Properties Realized with Liquid-Crystalline Ligands. Small 2019, 15 (37), 1902807 10.1002/smll.201902807. 31348618

[ref44] Zep A.; Wojcik M. M.; Lewandowski W.; Sitkowska K.; Prominski A.; Mieczkowski J.; Pociecha D.; Gorecka E. Phototunable Liquid-Crystalline Phases Made of Nanoparticles. Angew. Chem. 2014, 126 (50), 13945–13948. 10.1002/ange.201407497. 25297852

[ref45] Goerlitzer E. S. A.; Puri A. S.; Moses J. J.; Poulikakos L. V.; Vogel N. The Beginner’s Guide to Chiral Plasmonics: Mostly Harmless Theory and the Design of Large-Area Substrates. Adv. Opt. Mater. 2021, 9 (16), 2100378–2100378. 10.1002/adom.202100378.

[ref46] Dietrich K.; Lehr D.; Helgert C.; Tünnermann A.; Kley E. B. Circular Dichroism from Chiral Nanomaterial Fabricated by On-Edge Lithography. Adv. Mater. 2012, 24 (44), OP321–OP325. 10.1002/adma.201203424. 23042699

[ref47] Fredriksson H.; Alaverdyan Y.; Dmitriev A.; Langhammer C.; Sutherland D. S.; Zäch M.; Kasemo B. Hole-Mask Colloidal Lithography. Adv. Mater. 2007, 19 (23), 4297–4302. 10.1002/adma.200700680.

[ref48] Niori T.; Adachi S.; Watanabe J. Smectic Mesophase Properties of Dimeric Compounds. 1. Dimeric Compounds Based on the Mesogenic Azobenzene Unit. Liq. Cryst. 1995, 19 (1), 139–148. 10.1080/02678299508036731.

[ref49] Le K. V.; Takezoe H.; Araoka F. Chiral Superstructure Mesophases of Achiral Bent-Shaped Molecules – Hierarchical Chirality Amplification and Physical Properties. Adv. Mater. 2017, 29 (25), 1602737 10.1002/adma.201602737. 27966798

[ref50] Takanishi Y.; Takezoe H.; Fukuda A.; Watanabe J. Visual Observation of Dispirations in Liquid Crystals. Phys. Rev. B 1992, 45 (14), 7684–7689. 10.1103/PhysRevB.45.7684. 10000575

[ref51] Shadpour S.; Nemati A.; Salamończyk M.; Prévôt M. E.; Liu J.; Boyd N. J.; Wilson M. R.; Zhu C.; Hegmann E.; Jákli A. I.; Hegmann T. Missing Link between Helical Nano- and Microfilaments in B4 Phase Bent-Core Liquid Crystals, and Deciphering Which Chiral Center Controls the Filament Handedness. Small 2020, 16 (4), 1905591 10.1002/smll.201905591. 31885139

[ref52] Shadpour S.; Nemati A.; Boyd N. J.; Li L.; Prévôt M. E.; Wakerlin S. L.; Vanegas J. P.; Salamończyk M.; Hegmann E.; Zhu C.; Wilson M. R.; Jákli A. I.; Hegmann T. Heliconical-Layered Nanocylinders (HLNCs)-Hierarchical Self-Assembly in a Unique B4 Phase Liquid Crystal Morphology. Mater. Horizons 2019, 6 (5), 959–968. 10.1039/C9MH00089E.

[ref53] Novotná V.; Hamplová V.; Lejček L.; Pociecha D.; Cigl M.; Fekete L.; Glogarová M.; Bednárová L.; Majewski P. W.; Gorecka E. Organic Nanotubes Created from Mesogenic Derivatives. Nanoscale Adv. 2019, 1 (8), 2835–2839. 10.1039/C9NA00175A. 36133609 PMC9418705

[ref54] Gorecka E.; Vaupotič N.; Zep A.; Pociecha D. From Sponges to Nanotubes: A Change of Nanocrystal Morphology for Acute-Angle Bent-Core Molecules. Angew. Chemie - Int. Ed. 2016, 55 (40), 12238–12242. 10.1002/anie.201604915. 27593198

[ref55] Hough L. E.; Jung H. T.; Krüerke D.; Heberling M. S.; Nakata M.; Jones C. D.; Chen D.; Link D. R.; Zasadzinski J.; Heppke G.; Rabe J. P.; Stocker W.; Körblova E.; Walba D. M.; Glaser M. A.; Clark N. A. Helical Nanofilament Phases. Science. 2009, 325 (5939), 456–460. 10.1126/science.1170027. 19628864

[ref56] Ryu S. H.; Kim H.; Lee S.; Cha Y. J.; Shin T. J.; Ahn H.; Korblova E.; Walba D. M.; Clark N. A.; Lee S. B.; Yoon D. K. Nucleation and Growth of a Helical Nanofilament (B4) Liquid-Crystal Phase Confined in Nanobowls. Soft Matter 2015, 11 (39), 7778–7782. 10.1039/C5SM01783A. 26313738

[ref57] Lewandowski W.; Vaupotič N.; Pociecha D.; Górecka E.; Liz-Marzán L. M. Chirality of Liquid Crystals Formed from Achiral Molecules Revealed by Resonant X-Ray Scattering. Adv. Mater. 2020, 32 (41), 1905591 10.1002/adma.201905591. 32529663

[ref58] Siegel D. P.; Kozlov M. M. The Gaussian Curvature Elastic Modulus of N-Monomethylated Dioleoylphosphatidylethanolamine: Relevance to Membrane Fusion and Lipid Phase Behavior. Biophys. J. 2004, 87 (1), 366–374. 10.1529/biophysj.104.040782. 15240471 PMC1304357

[ref59] Siegel D. P. The Gaussian Curvature Elastic Energy of Intermediates in Membrane Fusion. Biophys. J. 2008, 95 (11), 5200–5215. 10.1529/biophysj.108.140152. 18805927 PMC2586550

[ref60] Selinger J. V.; Spector M. S.; Schnur J. M. Theory of Self-Assembled Tubules and Helical Ribbons. J. Phys. Chem. B 2001, 105 (30), 7157–7167. 10.1021/jp010452d.

[ref61] Chen D.; Heberling M. S.; Nakata M.; Hough L. E.; MacLennan J. E.; Glaser M. A.; Korblova E.; Walba D. M.; Watanabe J.; Clark N. A. Structure of the B4 Liquid Crystal Phase near a Glass Surface. ChemPhysChem 2012, 13 (1), 155–159. 10.1002/cphc.201100589. 22162333

[ref62] Lewandowski W.; Szustakiewicz P.; Kowalska N.; Grzelak D.; Narushima T.; Góra M.; Bagiński M.; Pociecha D.; Okamoto H.; Liz-Marzán L. M. Supramolecular Chirality Synchronization in Thin Films of Plasmonic Nanocomposites. ACS Nano 2020, 14 (10), 12918–12928. 10.1021/acsnano.0c03964. 32886482 PMC7596782

[ref63] Cho E. N.; Zhitomirsky D.; Han G. G. D.; Liu Y.; Grossman J. C. Molecularly Engineered Azobenzene Derivatives for High Energy Density Solid-State Solar Thermal Fuels. ACS Appl. Mater. Interfaces 2017, 9 (10), 8679–8687. 10.1021/acsami.6b15018. 28234453

[ref64] Hough L. E.; Clark N. A. Layer-Scale Optical Chirality of Liquid-Crystalline Phases. Phys. Rev. Lett. 2005, 95 (10), 107802 10.1103/PhysRevLett.95.107802. 16196967

[ref65] Matraszek J.; Topnani N.; Vaupotič N.; Takezoe H.; Mieczkowski J.; Pociecha D.; Gorecka E. Monolayer Filaments versus Multilayer Stacking of Bent-Core Molecules. Angew. Chem. 2016, 128 (10), 3529–3533. 10.1002/ange.201510123. 26833945

[ref66] Han J.; You J.; Li X.; Duan P.; Liu M. Full-Color Tunable Circularly Polarized Luminescent Nanoassemblies of Achiral AIEgens in Confined Chiral Nanotubes. Adv. Mater. 2017, 29 (19), 1606503 10.1002/adma.201606503. 28295680

[ref67] Liu R.; Feng Z.; Cheng C.; Li H.; Liu J.; Wei J.; Yang Z. Active Regulation of Supramolecular Chirality through Integration of CdSe/CdS Nanorods for Strong and Tunable Circular Polarized Luminescence. J. Am. Chem. Soc. 2022, 144 (5), 2333–2342. 10.1021/jacs.1c12676. 35077177

[ref68] Liu J.; Molard Y.; Prévôt M. E.; Hegmann T. Highly Tunable Circularly Polarized Emission of an Aggregation-Induced Emission Dye Using Helical Nano- and Microfilaments as Supramolecular Chiral Templates. ACS Appl. Mater. Interfaces 2022, 14 (25), 29398–29411. 10.1021/acsami.2c05012. 35713169

[ref69] Wang Y.-J.; Jin Y.; Shi X.-Y.; Dong X.-Y.; Zang S.-Q. Achiral Copper Clusters Helically Confined in Self-Assembled Chiral Nanotubes Emitting Circularly Polarized Phosphorescence. Inorg. Chem. Front. 2022, 9, 3330–3334. 10.1039/D2QI00982J.

[ref70] Vila-Liarte D.; Kotov N. A.; Liz-Marzán L. M. Template-Assisted Self-Assembly of Achiral Plasmonic Nanoparticles into Chiral Structures. Chem. Sci. 2022, 13 (3), 595–610. 10.1039/D1SC03327A. 35173926 PMC8768870

[ref71] Gong Y.; Cao Z.; Zhang Z.; Liu R.; Zhang F.; Wei J.; Yang Z. Chirality Inversion in Self-Assembled Nanocomposites Directed by Curvature-Mediated Interactions. Angew. Chemie - Int. Ed. 2022, 61 (10), e202117406 10.1002/anie.202117406. 34981650

[ref72] Chen Y.; Wang X. Novel Phase-Transfer Preparation of Monodisperse Silver and Gold Nanoparticles at Room Temperature. Mater. Lett. 2008, 62 (15), 2215–2218. 10.1016/j.matlet.2007.11.050.

[ref73] Peng S.; Lee Y.; Wang C.; Yin H.; Dai S.; Sun S. A Facile Synthesis of Monodisperse Au Nanoparticles and Their Catalysis of CO Oxidation. Nano Res. 2008, 1 (3), 229–234. 10.1007/s12274-008-8026-3.

[ref74] Gansel J. K.; Thiel M.; Rill M. S.; Decker M.; Bade K.; Saile V.; Von Freymann G.; Linden S.; Wegener M. Gold Helix Photonic Metamaterial as Broadband Circular Polarizer. Science. 2009, 325 (5947), 1513–1515. 10.1126/science.1177031. 19696310

[ref75] Namgung S. D.; Kim R. M.; Lim Y. C.; Lee J. W.; Cho N. H.; Kim H.; Huh J. S.; Rhee H.; Nah S.; Song M. K.; Kwon J. Y.; Nam K. T. Circularly Polarized Light-Sensitive, Hot Electron Transistor with Chiral Plasmonic Nanoparticles. Nat. Commun. 2022, 13 (1), 5081 10.1038/s41467-022-32721-2. 36038547 PMC9424280

[ref76] Bagiński M.; Tomczyk E.; Vetter A.; Suryadharma R. N. S.; Rockstuhl C.; Lewandowski W. Achieving Highly Stable, Reversibly Reconfigurable Plasmonic Nanocrystal Superlattices through the Use of Semifluorinated Surface Ligands. Chem. Mater. 2018, 30 (22), 8201–8210. 10.1021/acs.chemmater.8b03331.

