# Supplementary material for: Light-Responsive Supramolecular Nanotubes-Based Chiral Plasmonic Assemblies
Source: ACS Nano. 2023 Mar 10;17(6):5548–60. doi: 10.1021/acsnano.2c10955 (PMC10062029; doi:10.1021/acsnano.2c10955)
Supplement: Supplementary file 1 — nn2c10955_si_001.pdf [file nn2c10955_si_001.pdf]

Supporting Information:

Light-responsive supramolecular nanotubes-based chiral plasmonic assemblies

Agnieszka Jedrych, Mateusz Pawlak, Ewa Gorecka,

Wiktor Lewandowski, and Michal Maksymilian Wojcik**

Supporting Figures:

Figure S1: NMR analysis of 12OAzo5AzoO12.

Figure S2. ^{1}H NMR spectra of UV irradiated 12OAzo5AzoO12.

Figure S3. Further analysis of 12OAzo5AzoO12 molecular structure.

Figure S4. Structural investigation of 12OAzo5AzoO12.

Figure S5. Nanotubes formation by slow evaporation.

Figure S6. 12OAzo5AzoO12 film obtained by thermal quenching from the isotropic phase.

Figure S7. The influence of cooling speed on 12OAzo5AzoO12 nanotubes formation.

Figure S8. The lack of influence of heating rate on 12OAzo5AzoO12 nanotubes formation.

Figure S9. The lack of influence of substrate type on 12OAzo5AzoO12 nanotubes formation.

Figure S10. 12OAzo5AzoO12 in a liquid crystalline cell.

Figure S11. Correlated: polarizing optical microcopy (POM), UV-Vis, circular dichroism (CD) and SEM analysis.

Figure S12. Analysis of photoswitching of 12OAzo5AzoO12 films (UV and Vis irradiation).

Figure S13. Analysis of the symmetry of heat annealed thin film of Au4L_1.

Figure S14. The histogram of Au8L_1L_{AZO} nanoparticle diameters.

Figure S15. Structural and optical analysis of 12OAzo5AzoO12/Au8L_1 composites evidencing low chemical compatibility.

Figure S16. Synthetic route for the preparation of L_{AZO} compound.

Figure S17. Structural characterization of thin films of Au8L_1L_{AZO} with and without UV irradiation.

Figure S18. Structural analysis of 12OAzo5AzoO12/Au4L_1 composite films with varied amount of nanoparticles.

Figure S19. The impact of cooling rate on filling factor of organic nanotubes with Au4L_1 nanoparticles in 12OAzo5AzoO12/Au4L_1 composite films.

Figure S20. Small angle XRD measurements 12OAzo5AzoO12/Au4L_1 composites with 9 wt.% of nanoparticles.

Figure S21. Structural analysis of 12OAzo5AzoO12/Au8L_1L_{AZO} composites.

Figure S22. Structural analysis of 12OAzo5AzoO12/Au8L_1L_{AZO} composites with 10 wt.% of Au8L_1L_{AZO} NPs.

Figure S22. Structural analysis of 12OAzo5AzoO12/Au8L_1L_{AZO} composites with 10 wt.% of Au8L_1L_{AZO} NPs.

Figure S23. Detailed analysis center-to-center interparticle distances based on 3D, STEM based reconstruction of the sample structure.

Figure S24. A single, high-angle annular dark-field scanning transmission electron microscopy HAADF-STEM image of 12OAzo5AzoO12/Au8L_1L_{AZO} composite.

Figure S25. 3D reconstruction of Au8L_1L_{AZO} NPs organization in 12OAzo5AzoO12/ Au8L_1L_{AZO} composite.

Figure S26. UV-Vis analysis of Au4L_1 in the neat form and in composite.

Figure S27. UV-Vis analysis of Au8L_1L_{AZO} in the neat form and in composite.

Figure S28. An additional optical analysis of 12OAzo5AzoO12/Au4L_1 composite irradiated by UV at 80 ^{o}C, and then abruptly cooled to an ambient temperature.

Figure S29. An additional optical analysis of 12OAzo5AzoO12/Au4L_1 composite irradiated by UV at 80 ^{o}C, and then slowly cooled to an ambient temperature.

Figure S30. Small angle XRD measurements of 12OAzo5AzoO12/Au4L_1 composite before and irradiated by UV at 80 ^{o}C, and then slowly cooled to an ambient temperature. For comparison, 1D XRD diffractograms of thin films of Au4L_1 NPs is presented

Figure S31. Histogram of the diameter of nanotubules formed by nanoparticles for 12OAzo5AzoO12/Au4L_1 sample after UV irradiation at 70 ^{o}C and rapid quenching to an ambient temperature.

Figure S32. An additional optical analysis of 12OAzo5AzoO12/Au4L_1 composite irradiated by UV at 70°C, and then abruptly cooled to an ambient temperature.

Figure S33. Optical measurements of the 12OAzo5AzoO12 thin film illuminated with UV light at different temperatures.

Figure S34. Optical measurements of the 9 wt% of Au4L_1 in 12OAzo5AzoO12 thin film illuminated with UV light at different temperatures.

Supplementary Notes:

Note S1: Preparation of 12OAzo5AzoO12 thin films.

Note S2: Photoswitching of 12OAzo5AzoO12 thin films.

Note S3: Nanoparticle syntheses

Note S4: Introducing L_1 and L_{AZO} ligands to the surface of nanoparticles

Note S5: Preparation of 12OAzo5AzoO12/NP

Note S6: Studies of composites photoswitchability.

a)

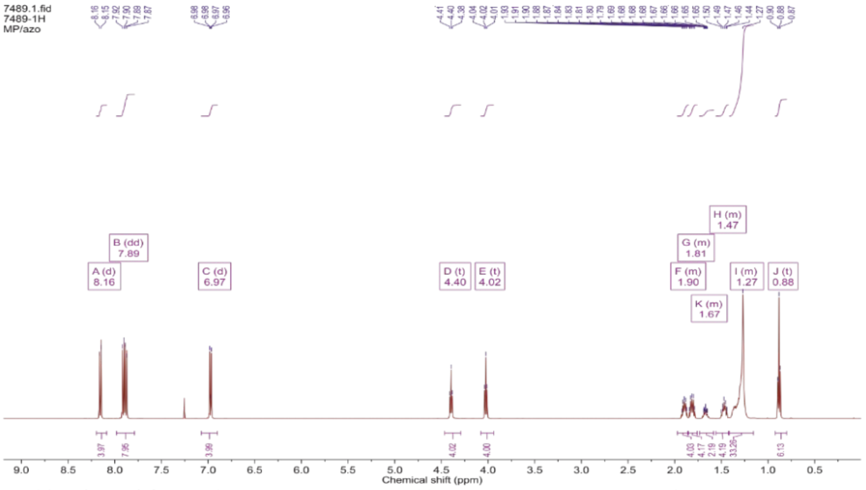

^{1}H NMR (500 MHz, Chloroform-*d*) δ 8.16 (d, *J* = 8.6 Hz, 4H), 7.89 (dd, *J* = 14.3, 8.8 Hz, 8H), 6.97 (d, *J* = 9.0 Hz, 4H), 4.40 (t, *J* = 6.5 Hz, 4H), 4.02 (t, *J* = 6.6 Hz, 4H), 1.97 – 1.85 (m, 4H), 1.86 – 1.76 (m, 4H), 1.74 – 1.58 (m, 2H), 1.56 – 1.42 (m, 4H), 1.41 – 1.16 (m, 33H), 0.88 (t, *J* = 6.9 Hz, 6H).

b)

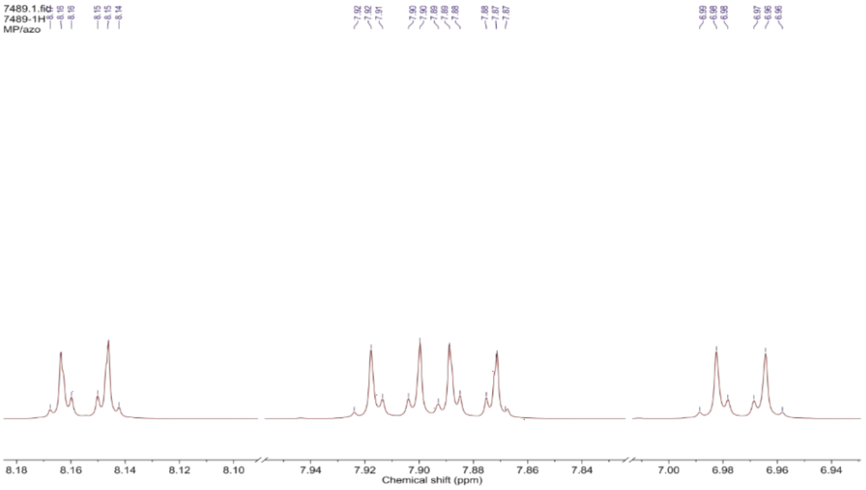

c)

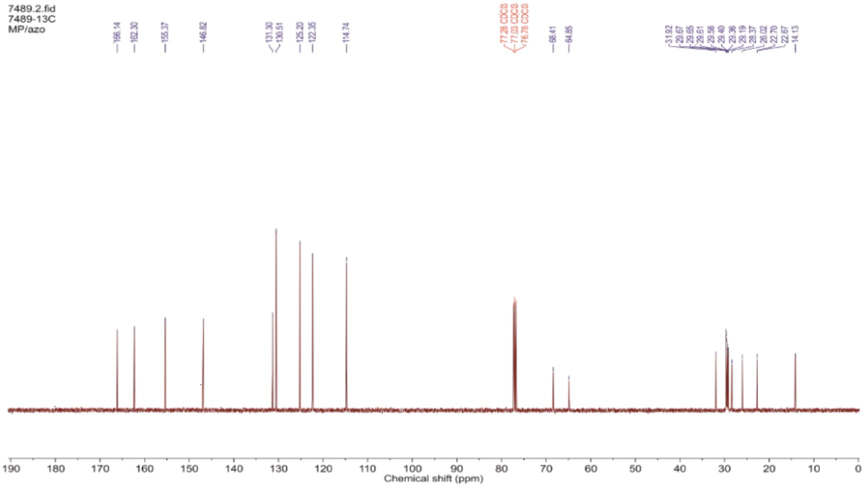

^{13}C NMR (126 MHz, $CDCl_3$) δ 166.14, 162.30, 155.37, 146.82, 131.30, 130.51, 125.20, 122.35, 114.74, 77.28, 77.03, 76.78, 68.41, 64.85, 31.92, 29.67, 29.65, 29.61, 29.58, 29.40, 29.36, 29.19, 28.37, 26.02, 22.70, 22.67, 14.13.

Figure S1. NMR analysis of 12OAzo5AzoO12. (a, b) ^{1}H NMR spectra with the magnification of the aromatic region. (c) ^{13}C NMR spectra.

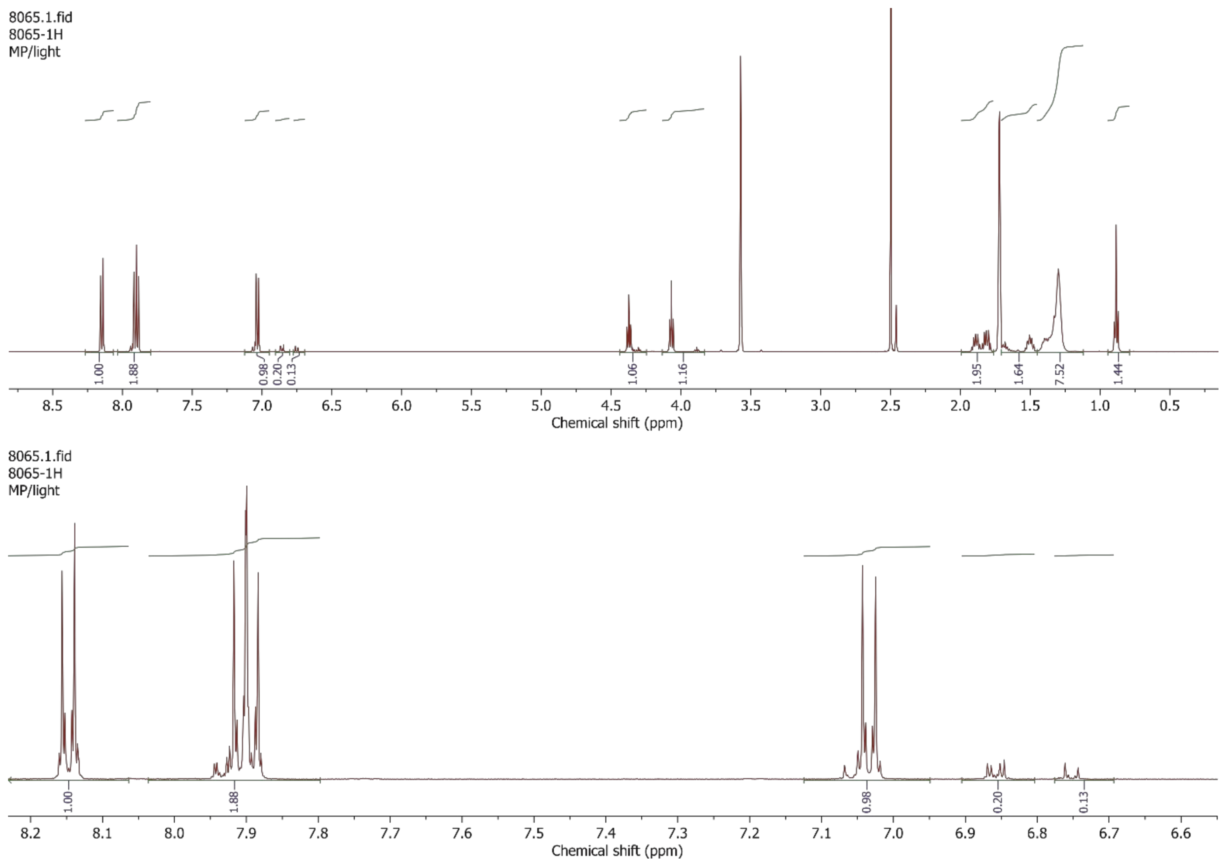

Figure S2. ^{1}H NMR spectra of UV irradiated tetrahydrofuran-d8 solution of 12OAzo5AzoO12 compound. The solution was irradiated with a 4W UV lamp at 365 nm for 30 minutes.

a)

Element	Mass percentage [%]
**Experimental**	
C	74,05 ± 0,01
H	8,56 ± 0,03
N	6,25 ± 0,05
**Calculated**	
C	74,29
H	8,61
N	6,3

b)

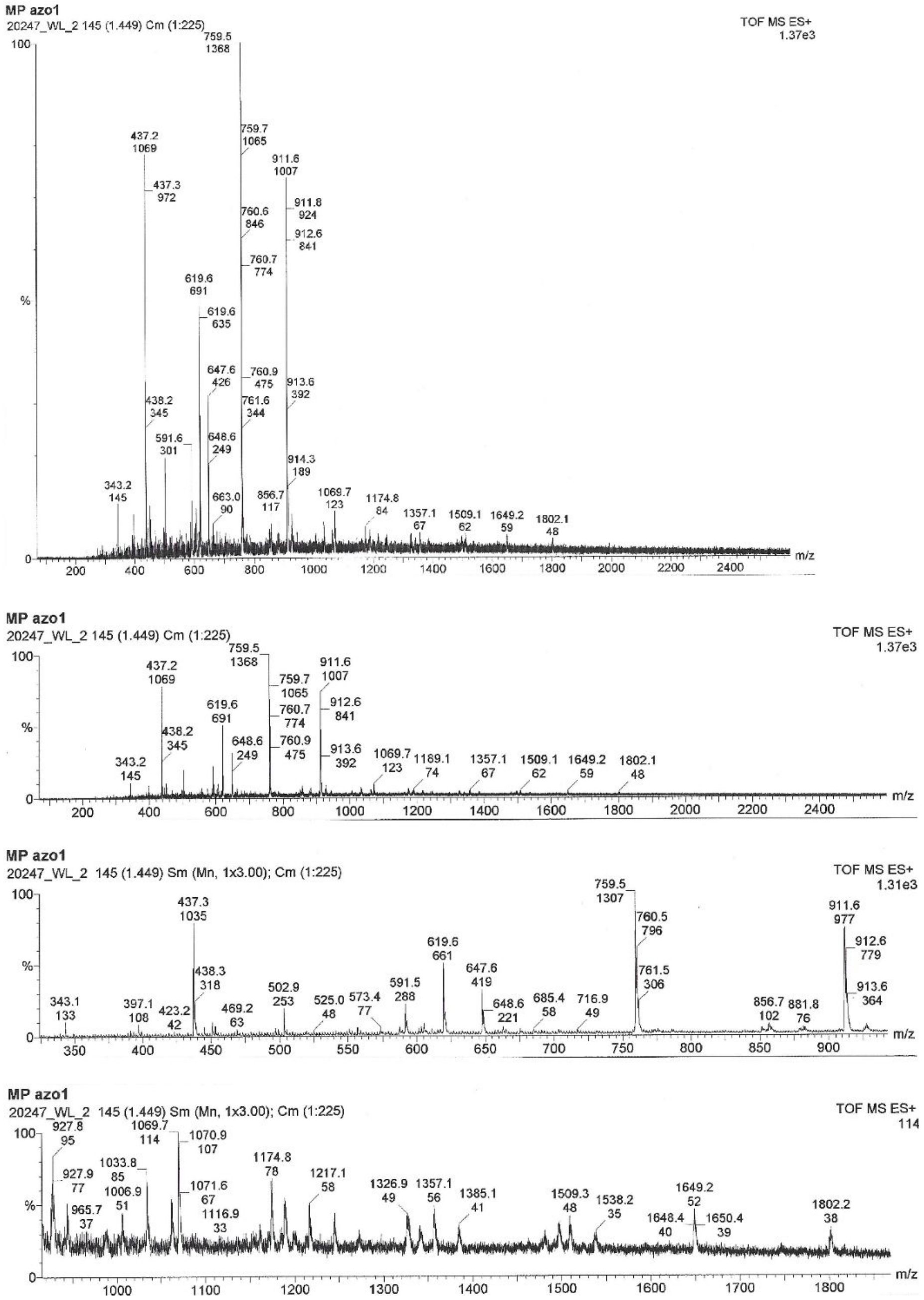

Figure S3. Further analysis of 12OAzo5AzoO12 molecular structure. (a) Elemental analysis. (b) Mass spectrometry analysis (TOF ES+).

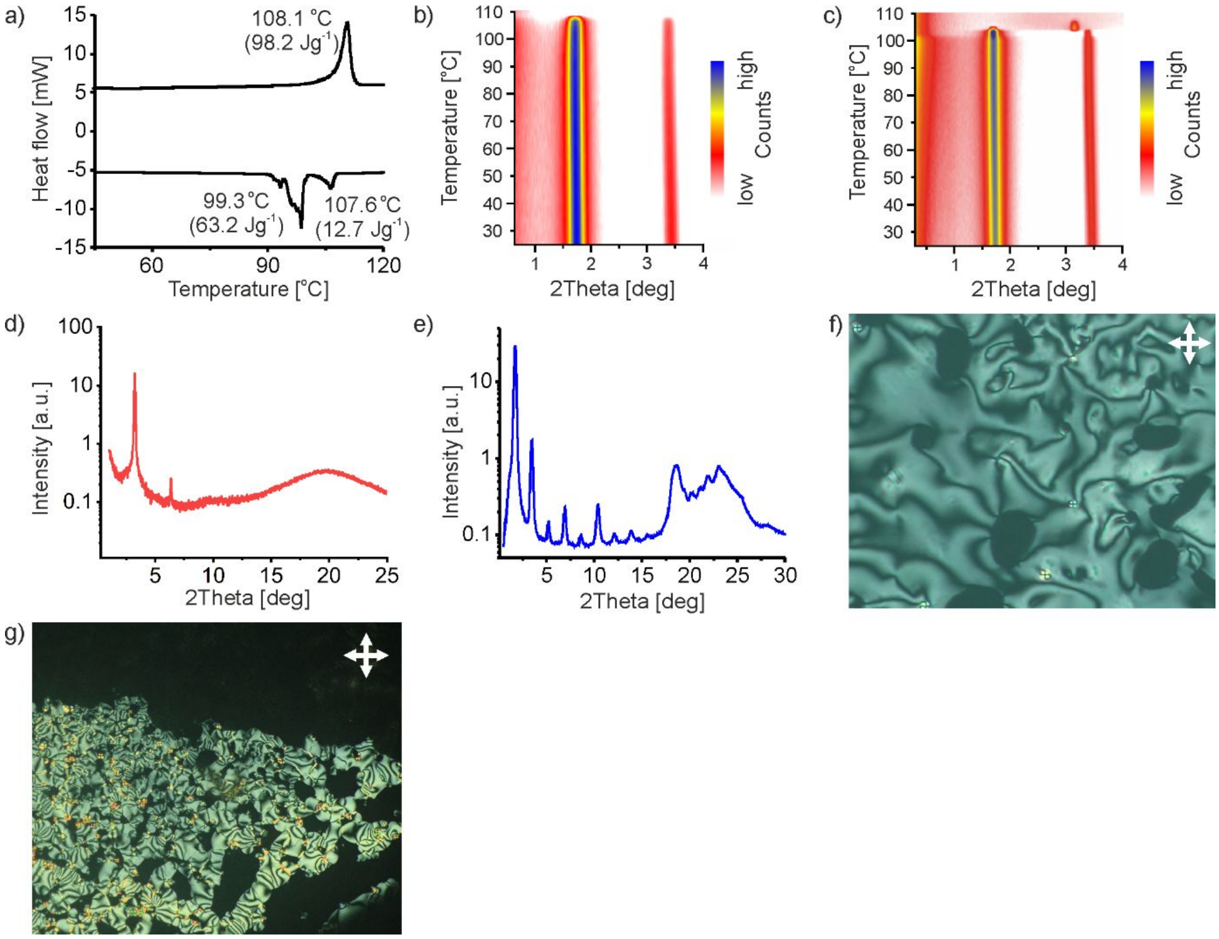

Figure S4. Structural investigation of 12OAzo5AzoO12. (a) A differential scanning calorimetry (DSC) curve. (b,c) Temperature evolution of SAXRD pattern obtained on heating and cooling 12OAzo5AzoO12 thin film, respectively; the investigated compound melts directly into the isotropic phase; phase transitions upon cooling give minute changes of the small-angle diffractograms, attesting the layered character of phases formed by 12OAzo5AzoO12 with almost constant inter-layer spacing. (d) 1D GADDS pattern collected at 110 °C, that is corresponding to the smectic phase. (e) 1D GADDS pattern collected at 30 °C, that is corresponding to Cry phase. (f, g) Polarizing optical microscope images confirming phase assignment to Sm (f) and Cry (g) phases.

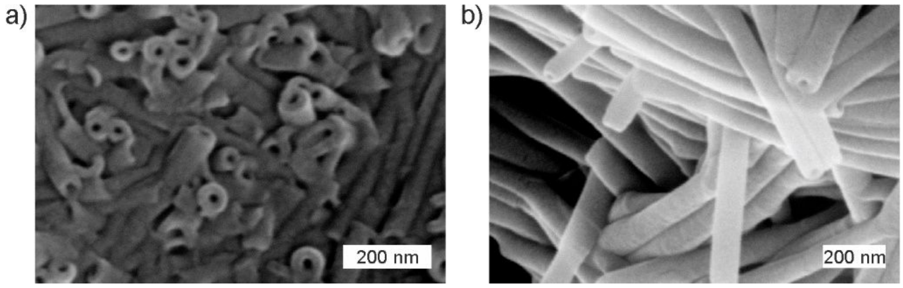

Figure S5. Nanotubes formation by slow evaporation of solvents attested by SEM images of 12OAzo5AzoO12 dropcasted on Si wafer, without thermal annealing. Samples were prepared by dropcasting from (a) tetrahydrofurane, and (b) toluene.

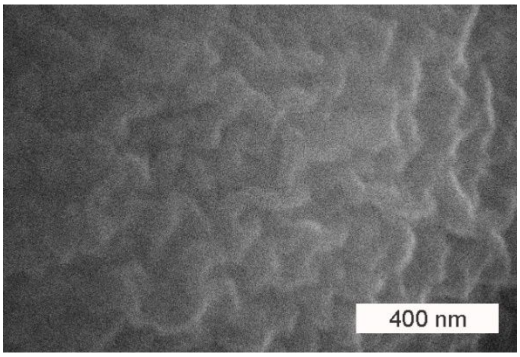

Figure S6. SEM image of an amorphous 12OAzo5AzoO12 film obtained by thermal quenching from the isotropic phase.

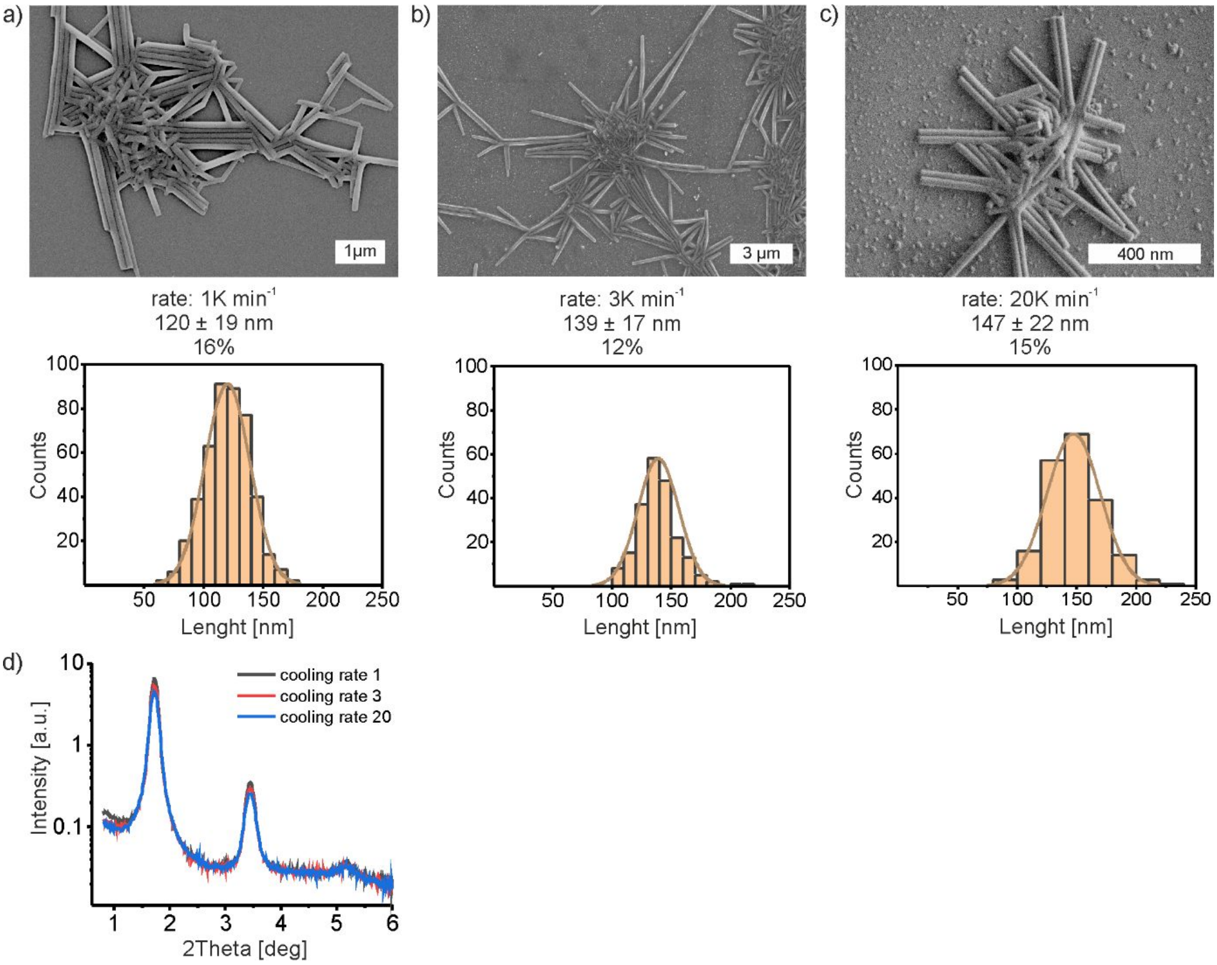

Figure S7. The influence of cooling speed on 12OAzo5AzoO12 nanotubes formation. (a-c) SEM images of samples prepared with different cooling speeds and graphs showing distribution of nanotubes diameters. (d) XRD diffractogram of 12OAzo5AzoO12 collected at 30°C, samples were cooled at different cooling rates.

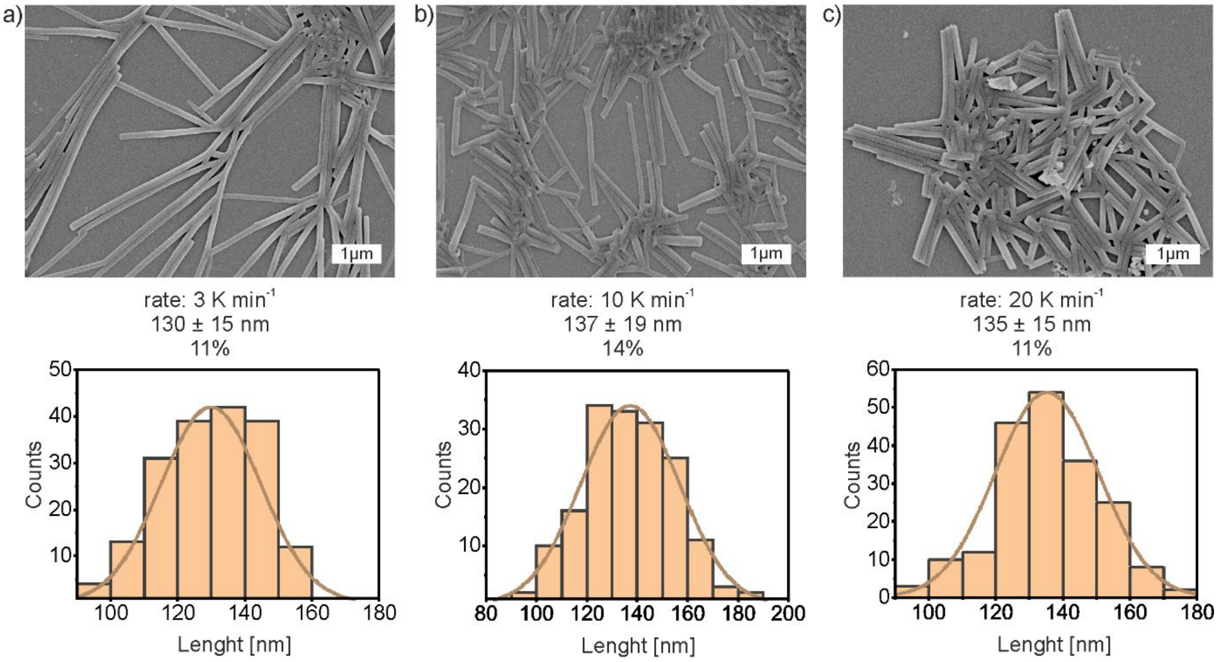

Figure S8. The lack of influence of heating rate on 12OAzo5AzoO12 nanotubes formation. SEM images of samples prepared with different heating speeds and graphs showing distribution of nanotubes diameters. Samples were heated at the rate (a) 1 K min^{-1}, (b) 3 K min^{-1}, and (c) 20 K min^{-1}, respectively.

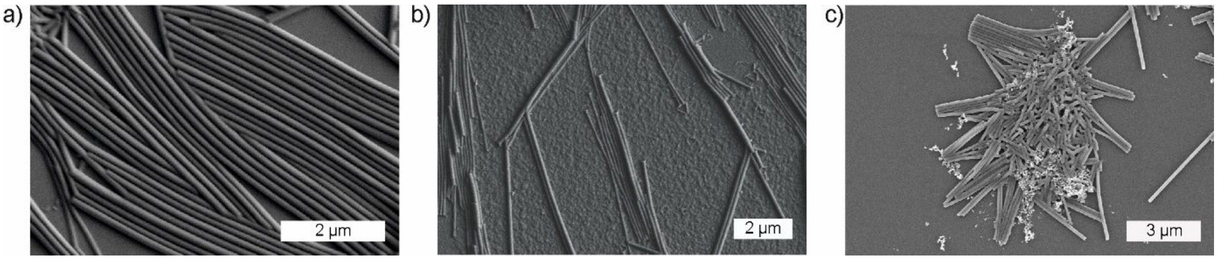

Figure S9. The lack of influence of substrate type on 12OAzo5AzoO12 nanotubes formation. SEM images of samples prepared by heat annealing on (a) Si, (b) glass, and (c) ITO coated glass substrates.

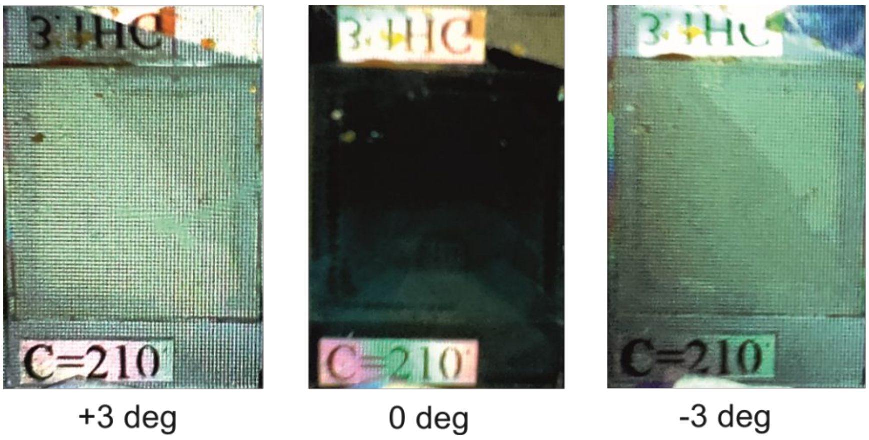

Figure S10. Polarizing optical microscopy investigation of 12OAzo5AzoO12 crystal phase at ambient temperature. 12OAzo5AzoO12 was infiltrated into a liquid crystalline cell. Size of obtained homochiral domains (bright/dark domains in images with 3 deg. decrossed polarizers) is on the order of centimeters.

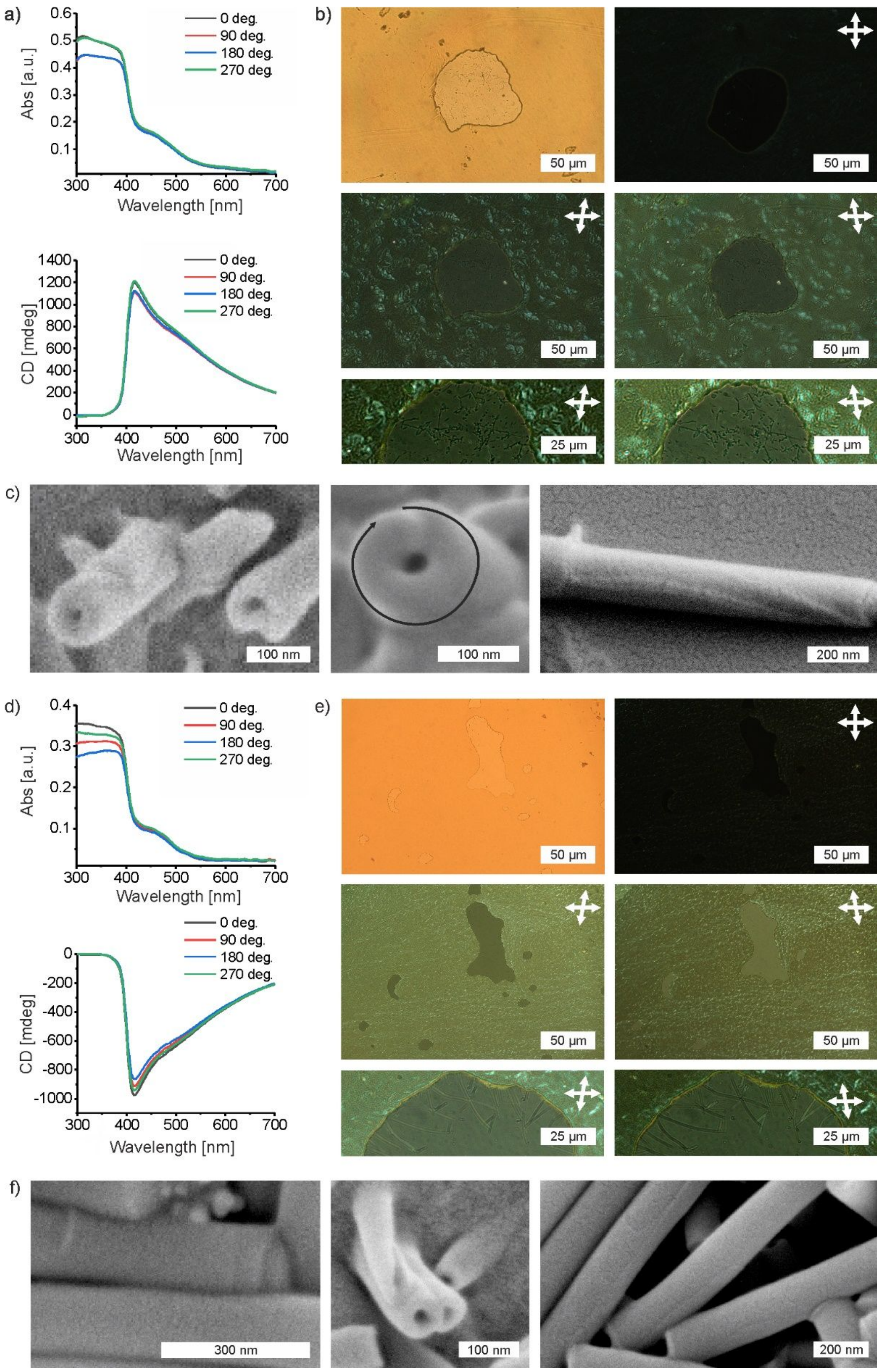

Figure S11. Correlated: polarizing optical microcopy (POM), UV-Vis, circular dichroism (CD) and SEM analysis of 12OAzo5AzoO12 domains in a thin film form. (a, d) UV-Vis and CD spectra of two homochiral domains having the opposite handedness. (b, e) POM investigation of domains analyzed in panels a, d. (c, f) SEM investigation of domains analyzed in panels a, d.

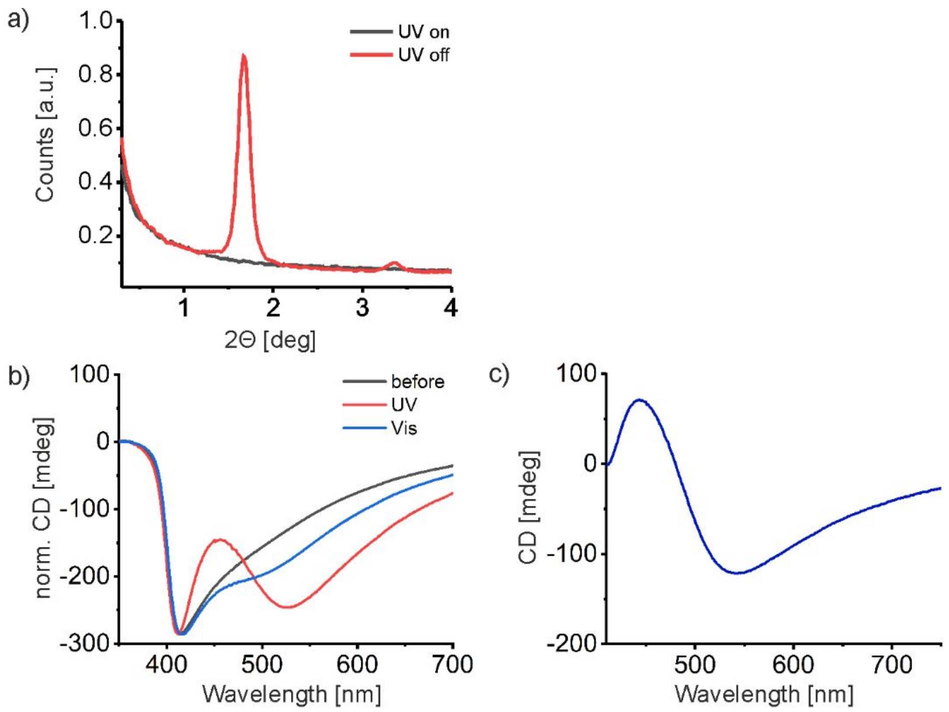

Figure S12. Analysis of photoswitching of 12OAzo5AzoO12 films (UV and Vis irradiation). (a) Disappearance of the main XRD peak was observed for sample after UV light irradiation in small angle region, attesting the loss of lamellar structure. (b) Normalized circular dichroism spectra of the native sample, after UV irradiation, and finally after Vis radiation; a weak, broad signal with Cotton characteristic also appeared at ~470 nm, that is in the spectral region of Z azobenzene absorption band. (c) The differential CD spectra of UV irradiated 12OAzo5AzoO12 vs. native sample.

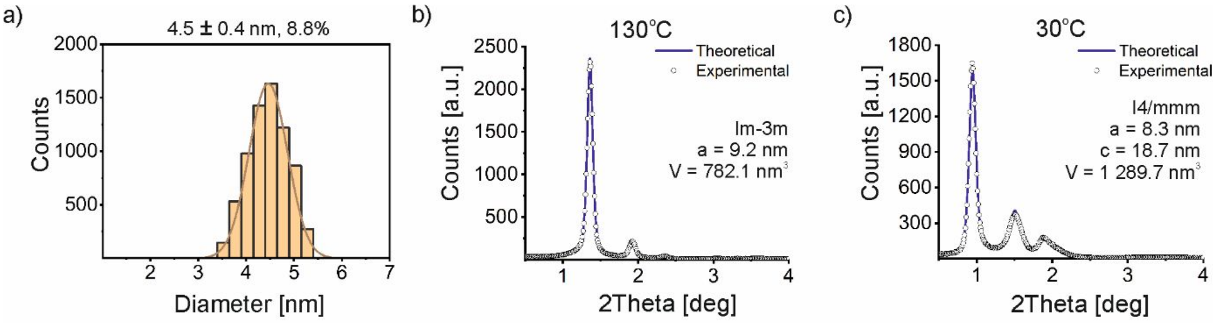

Figure S13. Analysis of the symmetry of heat annealed thin film of Au4L_1. (a) Histogram of NPs size (diameter). (b) 1D small angle XRD profile collected at 130 ^{o}C; the diffractogram was fitted using body centered cubic (BCC) symmetry, assuming a~9.2 nm unit cell dimension. (c) 1D small angle XRD profile collected at 30°C; the diffractogram was fitted using body centered tetragonal (BCT) symmetry, assuming a~8.3 and c~18.7 nm unit cell dimensions.

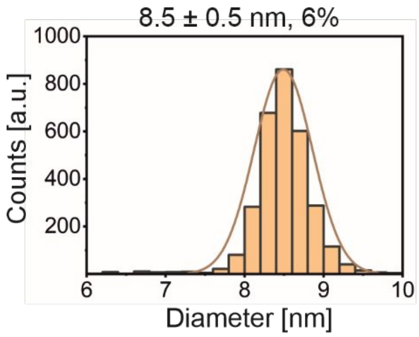

Figure S14. The histogram of $Au8L_1L_{AZO}$ nanoparticle diameters.

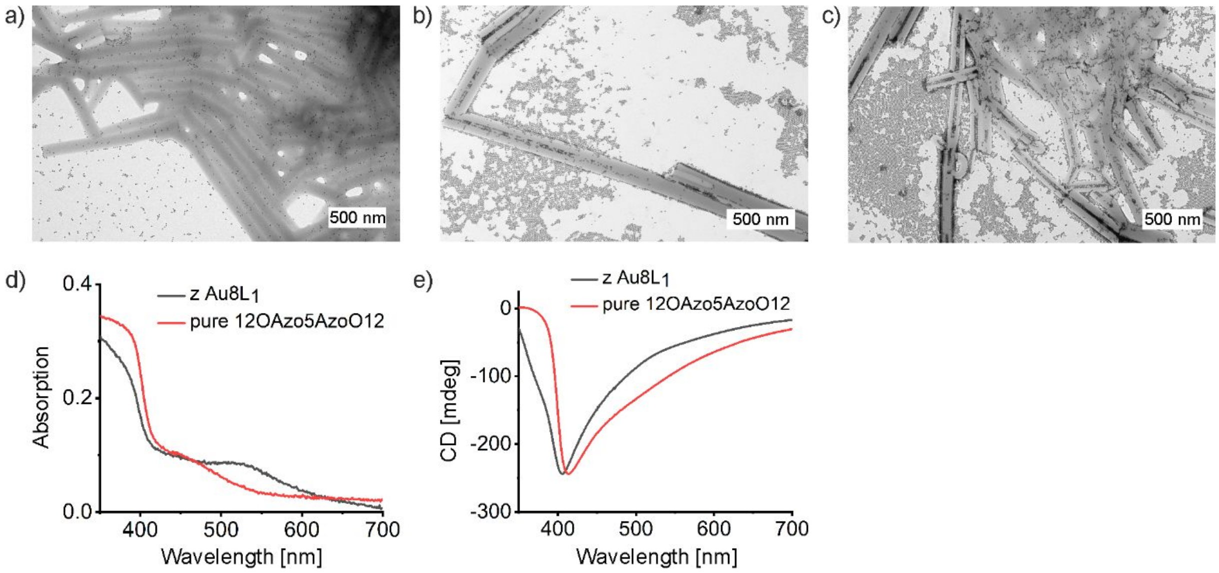

Figure S15. Structural and optical analysis of 12OAzo5AzoO12/$Au8L_1$ composites evidencing low chemical compatibility. (a-c) TEM images of composites obtained with lower and higher mass content of Au NPs. (d) UV-Vis spectra of pure 12OAzo5AzoO12 and in mixture with $Au8L_1$ NPs; plasmonic band is located at 530 nm. (e) Circular dichroism spectroscopy results, no additional band appeared in the plasmonic region.

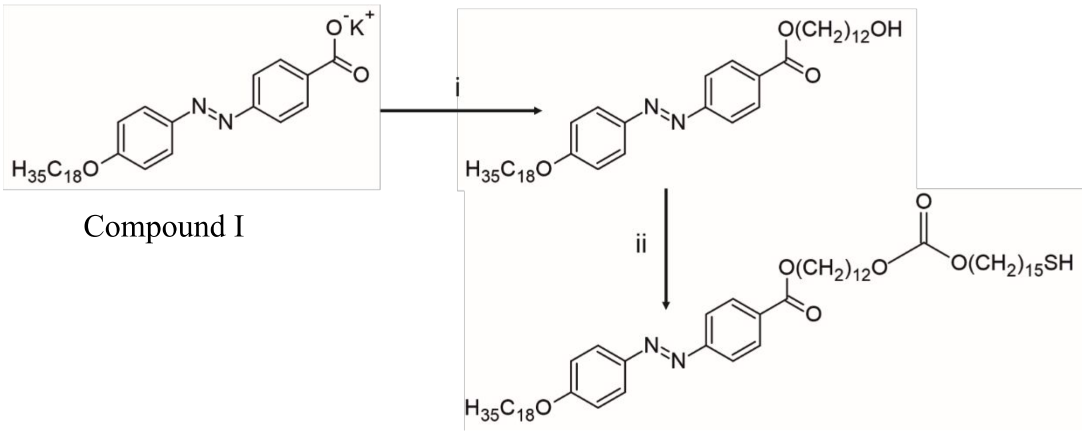

Figure S16. Synthetic route for the preparation of L_{AZO} compound. The starting material, compound I, is one of the semiproducts of synthesis of 12OAzo5AzoO12. Reagents and conditions: (i) a. oxalyl chloride, dimethylformamide, Dichloromethane, room temperature; b. 1,12-dodecanediol, pyridine, 4-(Dimethylamino)pyridine, tetrahydrofuran; (ii) 16-mercaptohexadecanoic acid, N,N′-Dicyclohexylcarbodiimide, tetrahydrofuran, 4-(Dimethylamino)pyridine, iced bath.

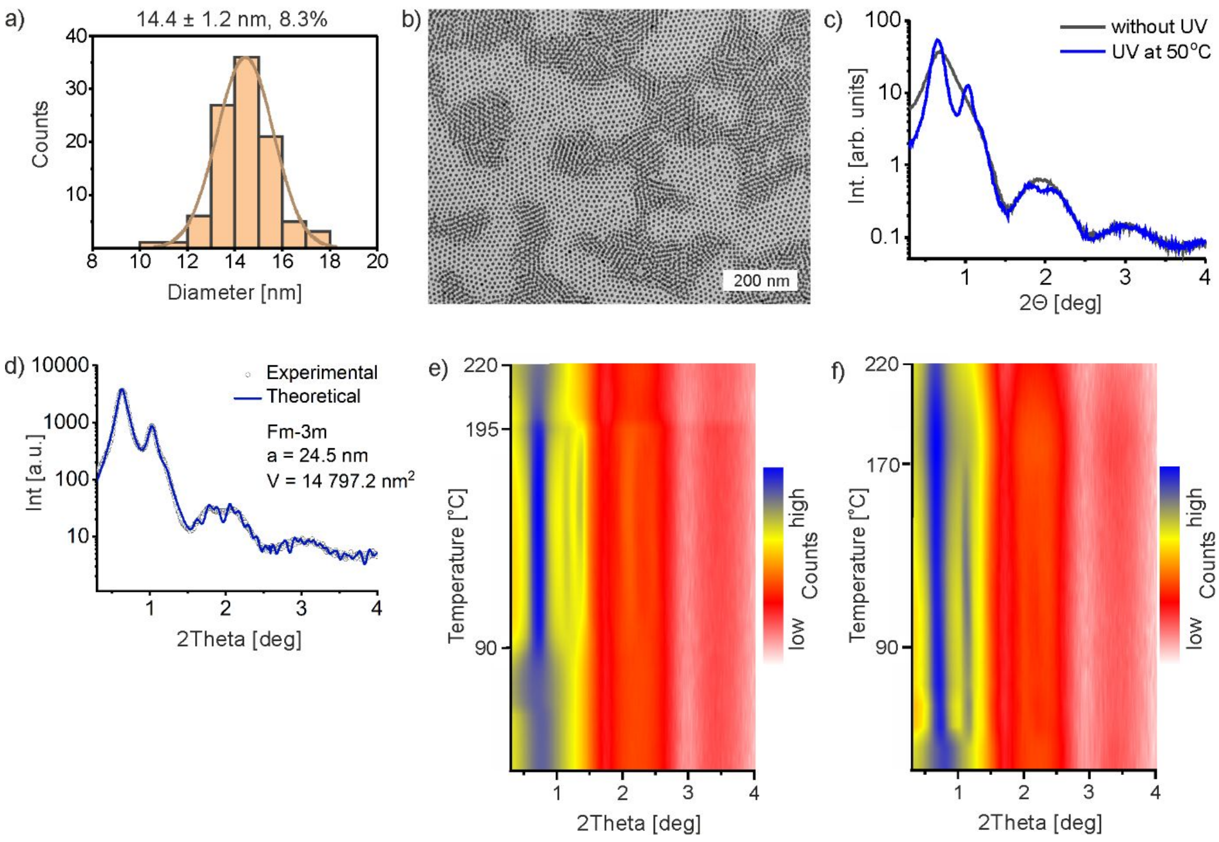

Figure S17. Structural characterization of thin films of $Au8L_1L_{AZO}$ with and without UV irradiation. (a) The average distance between cores of nanoparticles at room temperature, calculated based on the TEM images. (b) TEM image of $Au8L_1L_{AZO}$ irradiated by UV light at 50 ^{o}C; no obvious change in the packing of nanoparticles is observed. (c) Small angle XRD analysis of samples with and without UV irradiation; the sharpening of XRD peaks (growth of correlation length within nanopaticle superlattice) may originate from a E to Z conformation

change of the azo group, apparently leading to a more spherical shape of the organic shell. (d) 1D small angle XRD profile collected at 30 °C; the diffractogram was fitted using face centered cubic (FCC) symmetry, assuming a~24.5 nm unit cell dimension. (e, f) Temperature-dependent small angle XRD diffractograms of a thin film of nanoparticles (on a Kapton tape), without (e) and with UV radiation (f).

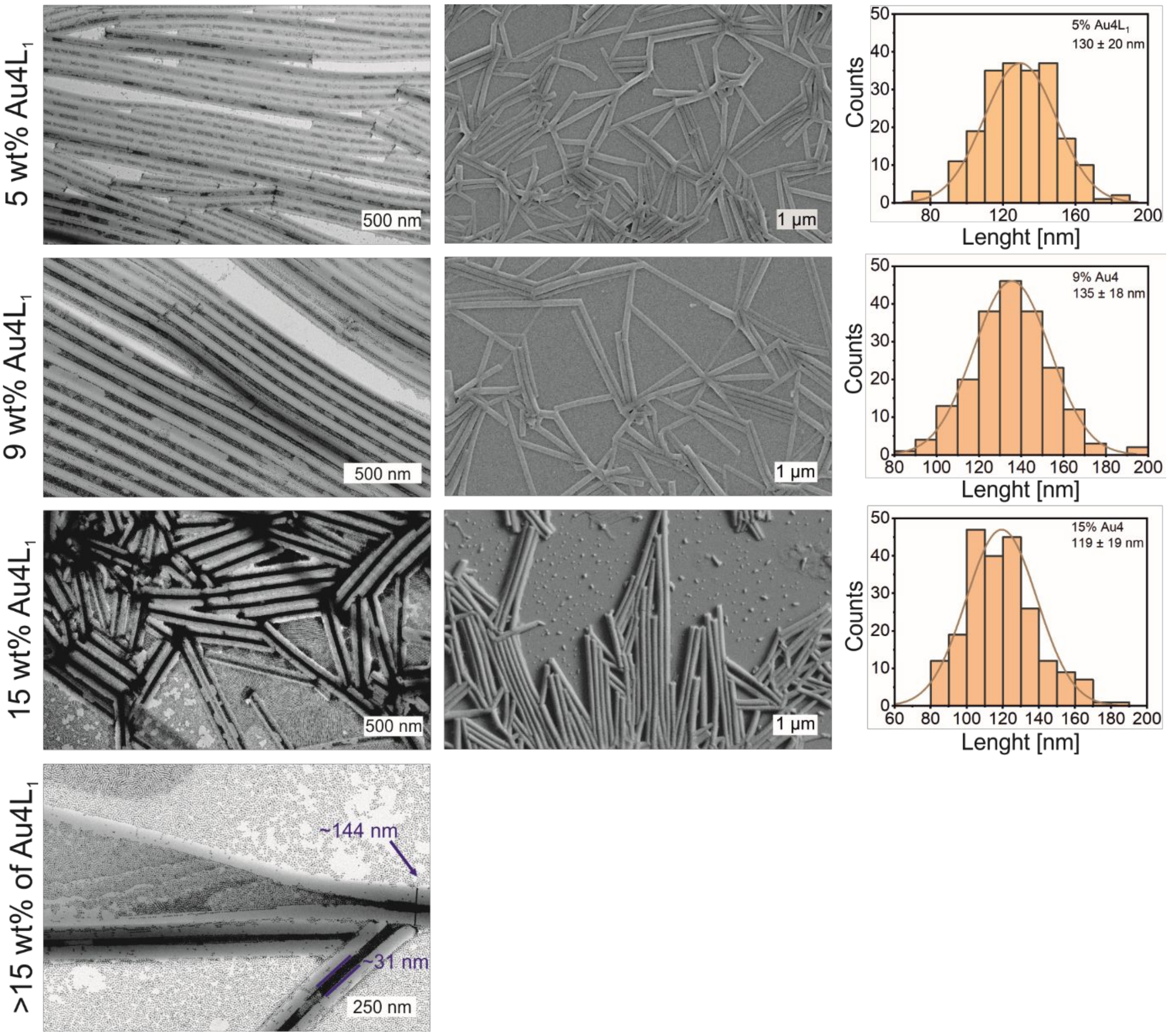

Figure S18. Structural analysis of 12OAzo5AzoO12/Au4L$_1$ composite films with varied amount of nanoparticles. These results attest that nanoparticles interacting with the matrix through ligands can affect twisting of the molecular organic layers into nanotubes. A partially unfolded organic nanotube (bottom, left) was found with an outer diameter of ~144 nm, and inner diameter of ~31 nm.

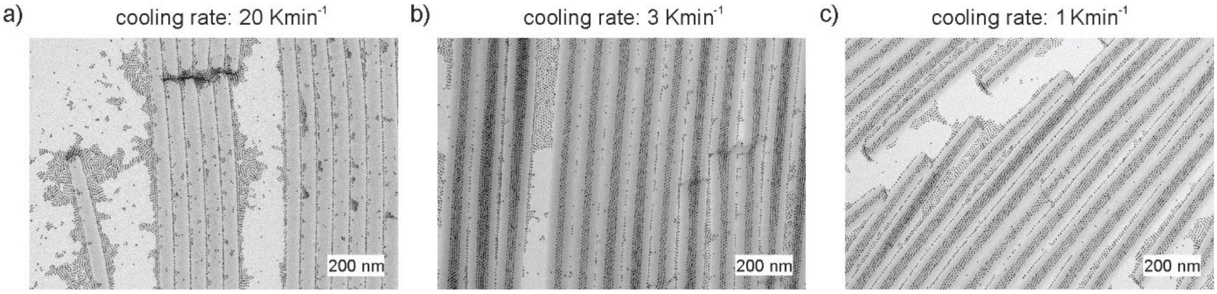

Figure S19. The impact of cooling rate on filling factor of organic nanotubes with $Au4L_1$ nanoparticles in $12OAzo5AzoO12/Au4L_1$ composite films.

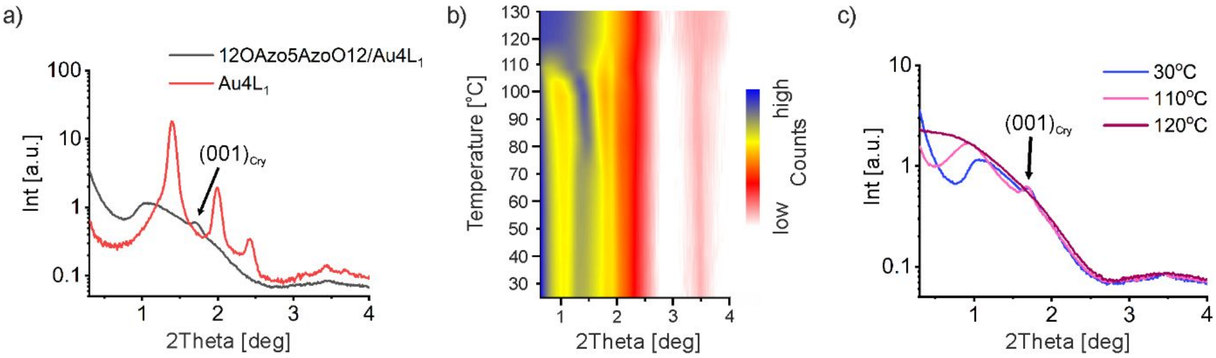

Figure S20. Small angle XRD measurements $12OAzo5AzoO12/Au4L_1$ composites with 9 wt.% of nanoparticles. (a) The comparison of 1D XRD diffractograms of thin films of $Au4L_1$ NPs and the composite; the (100) peak of pure 12OAzo5AzoO12 is visible; the absence of peaks characterisitc to NP films suggests strong interaction of nanoparticles with template. (b) Temperature-dependent small angle XRD diffractogram in a thin film on a Kapton tape; the sample is stable up to 115°C after which only broad scattering is observed. (c) The comparison of 1D XRD diffractograms of the tested sample at three temperatures – 30 °C, 110 °C and 120 °C, corresponding to 3 different phases of the compound.

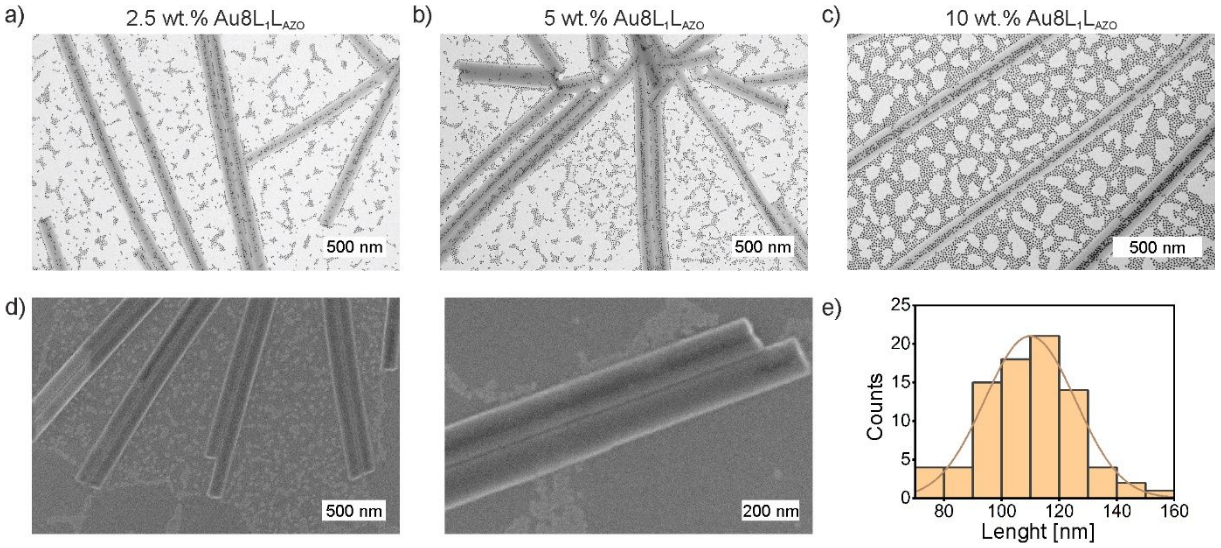

Figure S21. Structural analysis of 12OAzo5AzoO12/Au8L_1L_{AZO} composites. (a-c) TEM images of composites with varied amount of NPs. (d) SEM images of 12OAzo5AzoO12/Au8L_1L_{AZO} composite with 10 wt.% of Au8L_1L_{AZO}. (e) Histogram of nanotubes size, based on SEM images shown in (d).

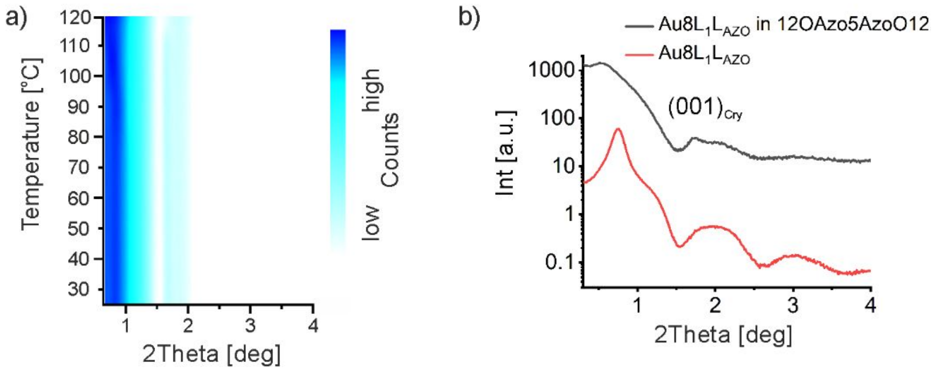

Figure S22. Structural analysis of 12OAzo5AzoO12/Au8L_1L_{AZO} composites with 10 wt.% of Au8L_1L_{AZO} NPs. (a) Temperature-dependent small angle XRD diffractogram in a thin film; the sample was prepared on a Kapton tape; the sample is stable up to 120°C. (b) 1D XRD diffractogram of the sample; the (100) peak characteristic to pure 12OAzo5AzoO12 is visible; the absence of peaks characterisitc to NP films suggests strong interaction of nanoparticles with template.

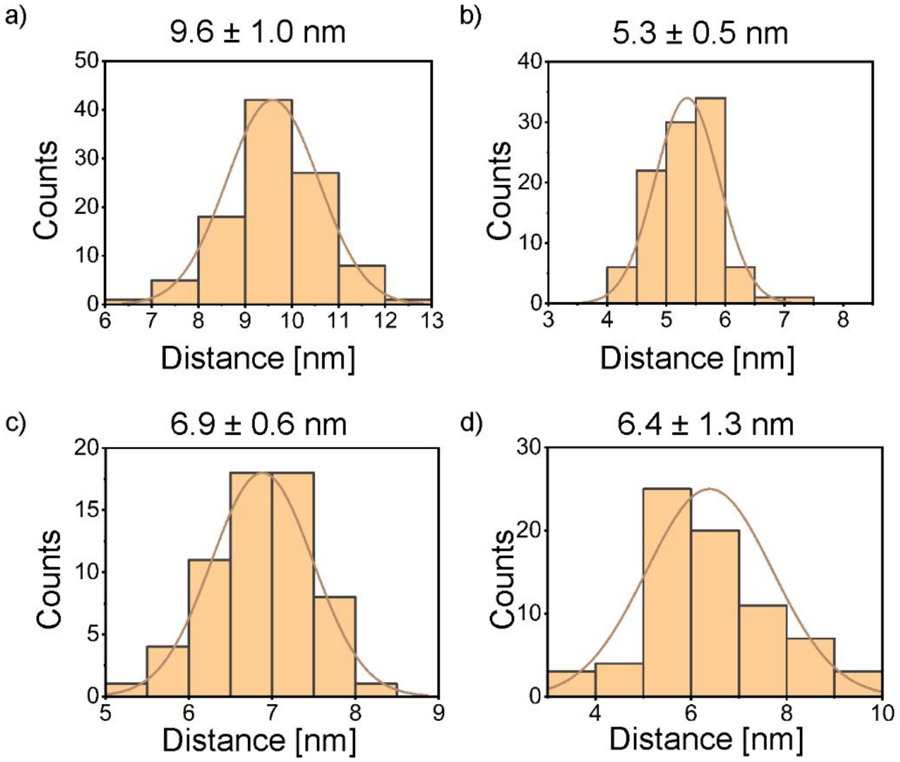

Figure S23. Detailed analysis center-to-center interparticle distances based on 3D, STEM based reconstruction of the sample structure. (a) Interlayer and (b) intralayer distances of nanoparticles forming assemblies outside the nanotubes. (c, d) the same for nanoparticles forming assemblies outside the nanotubes.

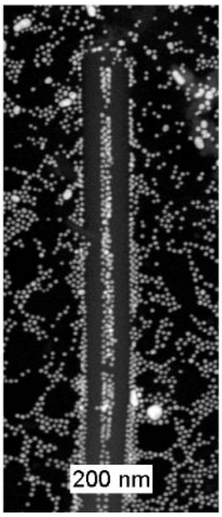

Figure S24. A single, high-angle annular dark-field scanning transmission electron microscopy HAADF-STEM image of 12OAzo5AzoO12/Au8L_1L_{AZO} composite.

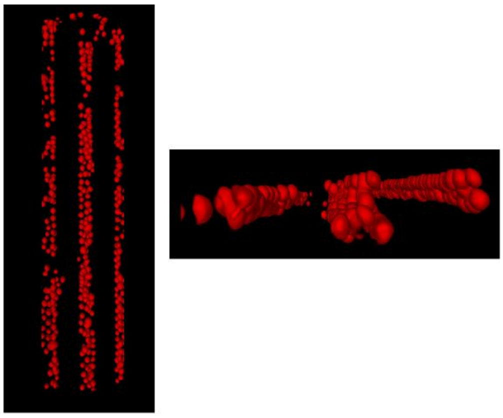

Figure S25. 3D reconstruction of $Au8L_1L_{AZO}$ NPs organization in 12OAzo5AzoO12/ $Au8L_1L_{AZO}$ composite. In analogy to 12OAzo5AzoO12/$Au4L_1$ results shown in the main text in Figure 5 nanoparticles form two types of assemblies: inside and outside of nanotubes.

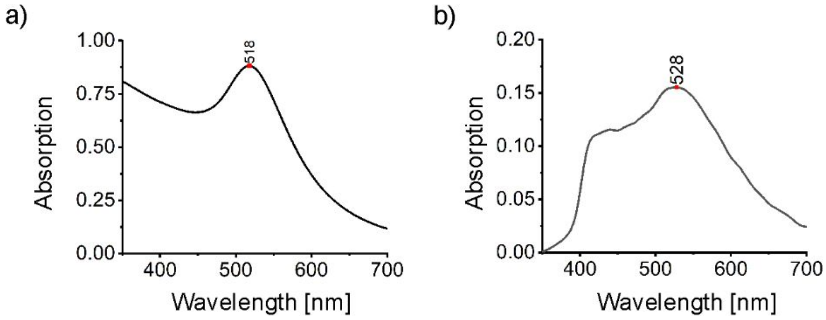

Figure S26. UV-Vis analysis of $Au4L_1$ in the neat form and in composite. (a) Dispersion in toluene, plasmonic band is centered at 518 nm. (b) Differential absorption spectra (12OAzo5AzoO12/$Au4L_1$ with 9 wt% of NPs vs. pure 12OAzo5AzoO12); the plasmonic band is shifted toward longer wavelengths – 528 nm.

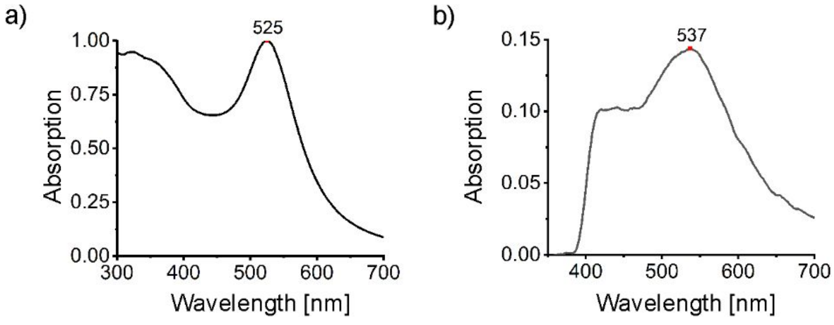

Figure S27. UV-Vis analysis of $Au8L_1L_{AZO}$ in the neat form and in composite. (a) Dispersion in toluene, plasmonic band is centered at 525 nm. (b) Differential absorption spectra (12OAzo5AzoO12/$Au8L_1L_{AZO}$ with 10 wt% of NPs vs. pure 12OAzo5AzoO12); the plasmonic band is shifted toward longer wavelengths – 537 nm.

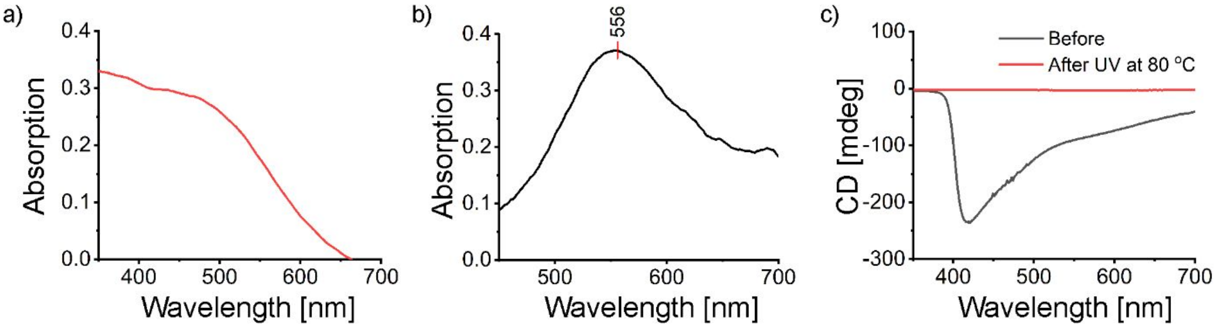

Figure S28. An additional optical analysis of 12OAzo5AzoO12/Au4L_1 composite irradiated by UV at 80°C, and then abruptly cooled to an ambient temperature. (a) UV-Vis spectra after UV irradiation. (b) Differential UV-Vis spectra of UV irradiated and non-irradiated sample. (c) CD spectra of UV irradiated and non-irradiated sample.

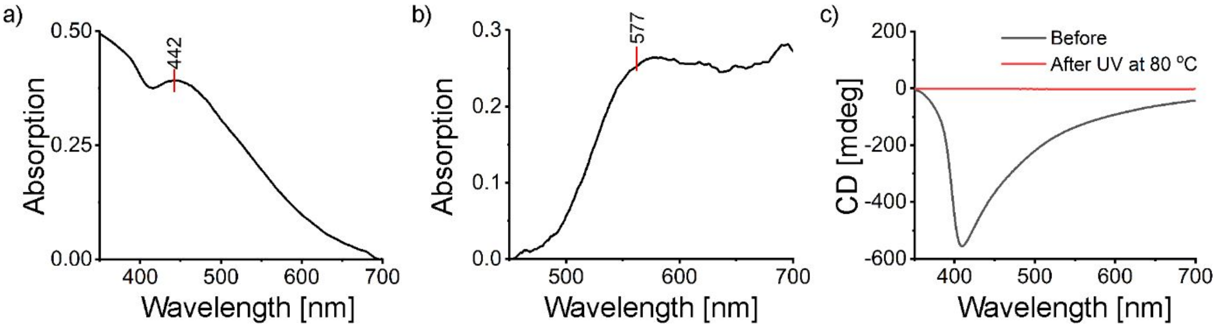

Figure S29. An additional optical analysis of 12OAzo5AzoO12/Au4L_1 composite irradiated by UV at 80°C, and then slowly cooled (3 K min^{-1}) to an ambient temperature. (a) UV-Vis spectra after UV irradiation. (b) Differential UV-Vis spectra of UV irradiated and non-irradiated sample. (c) CD spectra of UV irradiated and non-irradiated sample.

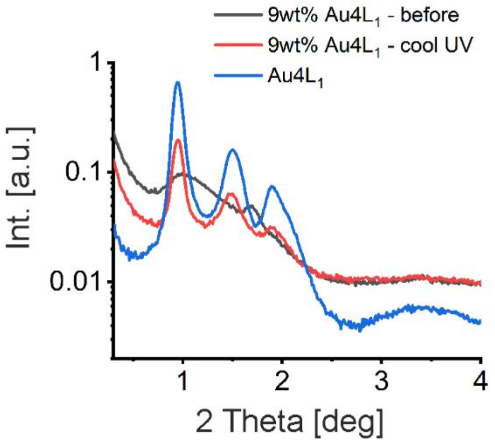

Figure S30. Small angle XRD measurements of 12OAzo5AzoO12/Au4L_1 composite before (black) and irradiated by UV at 80 ^{o}C, and then slowly cooled to an ambient temperature (red). For comparison, 1D XRD diffractogram of thin films of Au4L_1 NPs is presented (blue).

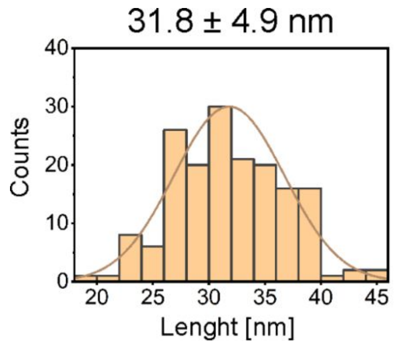

Figure S31. Histogram of the diameter of nanotubules formed by nanoparticles for 12OAzo5AzoO12/Au4L_1 sample after UV irradiation at 70 °C and rapid quenching to an ambient temperature.

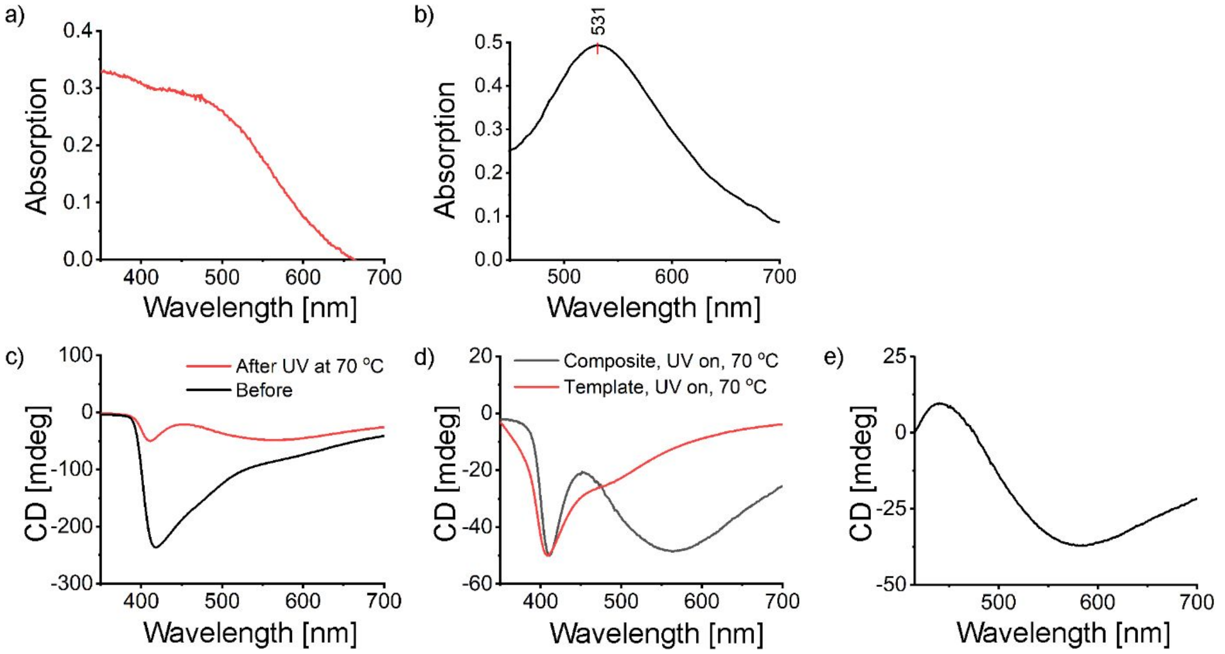

Figure S32. An additional optical analysis of 12OAzo5AzoO12/Au4L_1 composite irradiated by UV at 70°C, and then abruptly cooled to an ambient temperature. (a) UV-Vis spectra after UV irradiation. (b) Differential UV-Vis spectra of UV irradiated and non-irradiated sample. (c) CD spectra of UV irradiated and non-irradiated sample. (d) Differential CD spectra of UV irradiated samples (12OAzo5AzoO12/Au4L_1 with 9 wt% of NPs vs. pure 12OAzo5AzoO12). (e) Differential CD spectra of results presented in panel d.

SUPPLEMENTARY NOTES

Note S1: Preparation of 12OAzo5AzoO12 thin films.

12OAzo5AzoO12 was stored in a glove box under nitrogen atmosphere and protected from light. A small portion of the 12OAzo5AzoO12 compound (5.0 mg) was dissolved in 2.5 ml of THF. This solution was stored in brown glass vials. 77.4 µl of obtained solution was dropcasted on glass substrate or TEM grid or Kapton tape for CD, TEM or XRD measurements, respectively. Samples were heat annealed before measurements by melting the sample to the isotropic phase (~130°C) and slowly cooling it down to room temperature (heating rate: 20 K per minute, cooling rate: 3 K per minute). Analogous conditions were previously used for the fabrication of purely organic and composite thin films of morphologically chiral liquid crystals[1]. The temperature and cooling rates of the samples were precisely controlled by a heating stage (THMSG600, Linkam, UK).

We have chosen 3 K min^{-1} cooling rate as the standard protocol for the formation of thin films, due to few reasons:

- Out of the tested cooling rates this one yielded nanotubes with the lowest distribution of width
- Minimizing the time nanoparticles are at the elevated temperature when fabricating composite films with nanoparticles, while keeping the quality of the formed structures
- With this cooling rate it was possible to form centimeter size homochiral domains, enabling macroscopic measurements of chiroptical properties without using any chiral dopants.

Note S2: Photoswitching of 12OAzo5AzoO12 thin films.

Thin 12OAzo5AzoO12 film prepared on a glass substrate according to the Note S1. The samples were irradiated using a light source Hamamatsu L9588 Lightning Cure spot light source model LC8 equipped with interchangeable filters allowing for choosing UV (<380 nm passing band) or Vis illumination (>390 nm passing band). The effective power density at 365 nm is 4500 mW cm^{-2}.

The thin film of 12OAzo5AzoO12 compound is not photoswitchable at room temperature. In order to determine the optimal temperature necessary for photoswitching, UV-Vis and CD measurements of the sample illuminated with UV light at different temperatures were carried out (Figure S33). In these experiment, the sample was placed on a heating stage and its temperature was raised to a given temperature with a rate 20 K per minute. After 60s, the UV lamp was turned on. Illumination was continued without interruption while the sample was rapidly cooled to room temperature (cooling rate: 40 K per minute) and then directly used for measurements.

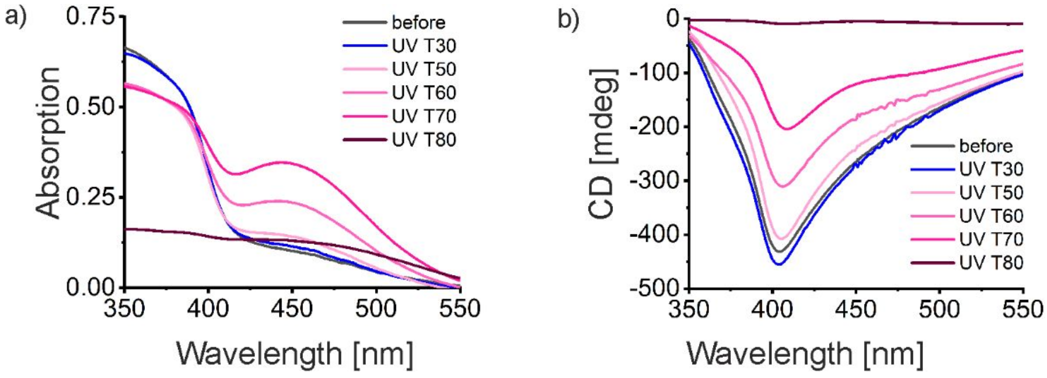

Figure S33. Optical measurements of the 12OAzo5AzoO12 thin film illuminated with UV light at different temperatures: a) UV-Vis and b) CD, respectively.

To fully understand the efficiency of switching, the fraction of Z isomer was approximated by taking the ratio of the absorption of π→π* and the initial E dominant film absorption ratio and subtracting it from 1 according to the method published by Grossman et al.[2]. For non-irradiated sample the intensity of absorption was 0.587 at 375 nm, which corresponds to π→π * transition. The detailed data for irradiated samples are presented below in Table S1.

$$Fraction\ of\ Z\ isomer = 1 - \frac{\pi \rightarrow \pi^*}{\pi \rightarrow \pi^*_{before}}$$

Table S1. The intensity of absorption at 375 nm for 12OAzo5AzoO12 thin film illuminated with UV light at different temperatures

Temperature [°C]	Absorption at 375 nm corresponding to π→π * transition	Fraction of Z isomer
30	0.585	0.004
50	0.518	0.117
60	0.519	0.117
70	0.519	0.117
80	0.156	0.734

The highest Z-isomer fractions reported by Grossmann et al. for thin film samples was 0.52. Here, 0.734 fraction was achieved for the thin film irradiated at 80 °C. Notably, although not all molecules are switched to the Z configuration, SEM convincingly proofs that already with this amount of Z isomer nanotubes are not formed. However, we are not able to quantify the presence of E/Z, Z/Z and E/E isomers of 12OAzo5AzoO12 in the mixture – possibly all are present.

We further confirmed structural switchability by SAXRD measurements. For this purpose temperature of the sample was constant (80 °C). The sample after thermal annealing on Kapton tape was alternately exposed to UV / Vis irradiation during 180s (time of one SAXRD measurement, Figure 2f). The disappearance of the main XRD peak, corresponding to the

formation of lamellar structure by 12OAzo5AzoO12 compound, when irradiated with UV, confirmed that the achieved level of switching was preventing molecular assembly into ordered structures.

The sample prepared for SEM investigation was prepared by heating to 80 °C, performing 10 cycles of UV / Vis switching for 60s, and then cooled to room temperature (cooling rate: 3 K per minute).

Note S3: Nanoparticle syntheses

Synthesis of 4.5 nm diameter gold nanoparticles (Au4)

Spherical gold nanoparticles with 4.5 nm of diameter were obtained according to a modified literature procedure[3]. Dodecylamine (3g, 16.19 mmol) was dissolved in cyclohexane (100 ml), then 12 ml of aqueous formaldehyde solution (37%) was added and vigorously stirred for 10 min. Organic phase was separated out and washed twice with water (2x30 ml). Next, an aqueous solution of tetrachloroauric acid (0.08 g $HAuCl_4$ in 20 ml of water) was added under vigorous stirring. After further stirring (40 min), cyclohexane phase was separated out by centrifugation. Next, 2 mL of dodecanethiol were added and the reaction mixture was stirred overnight at low rpm. A precipitate formed. The precipitate was centrifuged (10 min, 6000 rpm), and then 150 ml of acetone was added. A new precipitate was formed and centrifuged (10 min, 6000 rpm), collected and dissolved in a small amount of cyclohexane (10 ml). The precipitation procedure was repeated twice. Then, the obtained solution was again centrifuged (10 min, 8000 rpm), in order to get rid of insoluble aggregates. To get fractions of NPs with low size distribution, a fractionation process was performed. Namely, a small amount of ethanol was added to the cyclohexane NPs solution until turbidity appeared. The precipitate was centrifuged (10 min, 6000 rpm), and dissolved in a small amount of cyclohexane. A further portion of ethanol was added to the remaining supernatant. This process was repeated three additional times, yielding fractions containing smaller and smaller NPs. Fraction with the highest amount of particles was chosen for further work.

Synthesis of 8.5 nm diameter gold nanoparticles (Au8)

Spherical gold nanoparticles with 8.5 nm of diameter were obtained according to a modified literature procedure[4]. A solution of gold precursor was prepared by adding tetrachloroauric acid ($HAuCl_4{\cdot}3H_2O$, 0.1g) and 10 mL of oleylamine to 10 mL of tetralin. This dispersion was magnetically stirred in a glove box under nitrogen and cooled down to 10^oC. A reducing solution containing 0.5 mmol of tetrabutylammonium bromide (TBAB), 1 mL of tetralin, 1 mL of oleyamine was mixed by sonication and injected into the precursor solution. The reduction was instantaneously initiated and the solution changed color to a deep purple. The mixture was allowed to react at r.t. for 1 h before acetone (60 mL) was added to precipitate the Au NPs. The Au NPs were collected by centrifugation (8500 rpm, 8 min), washed with acetone and redispersed in hexane. To get fractions of NPs with slight size distribution, a fractionation process was performed as described for Au4 NPs.

Note S4: Intoducing L_1 and L_{AZO} ligands to the surface of nanoparticles

Liquid crystal-like ligands were introduced to the surface of nanoparticles using a modified literature method[5,6].

Preparation of Au4L_1 nanoparticles

To 5 mg of 4.5 nm diameter NPs (Au4) coated with dodecanethiol dissolved in 3 ml of cyclohexane, 5 mg of LC-like ligand (L_1) dissolved in 5 ml of toluene was added. The reaction mixture was slowly stirred overnight. Then, solvents were evaporated and the 2 ml of toluene were added, NPs were precipitated with 5 ml of ethanol and centrifuged (5 min, 6000 rpm). The supernatant containing unbound thiol ligands was removed and precipitate was dissolved in 2 ml of toluene. NPs were again precipitated by addition of 5 ml of ethanol and centrifuged. This washing procedure was repeated until no unbound ligand was presented on TLC plate. Finally, NPs were dissolved in toluene (2 ml, 2.5 mg mL^{-1}) and centrifuged (10 min, 5000 rpm) to remove any aggregates.

It has previously been reported that functionalization of nanoparticle's surface with dodecanethiol and LC-like ligands enables efficient formation of assemblies of metallic nanoparticles into liquid crystal template[1,7,8]. This type of coating has been shown to provide efficient mixing of nanoparticles with the matrix in the isotropic phase.

Preparation of Au8L_1 nanoparticles

In a typical procedure, 5 mg of Au8 coated with DDT was dissolved in 2.5 mL of hexane. Then, this solution was added to 2 mL of 2.5 mg mL^{-1} toluene solution of LC-like ligand L_1. The mixture was left at vortex with slow (100 rpm) stirring overnight. Then, 10 mL of ethanol was added, and the mixture was centrifuged for 5 min at 6000 rpm. Finally, the supernatant was discarded, precipitated nanoparticles were redissolved in toluene, and the precipitation procedure was repeated until no unbound ligand was presented on TLC plate.

Preparation of Au8L_1L_{AZO} nanoparticles

In a typical procedure, 5 mg of Au8 coated with DDT was dissolved in 2.5 mL of hexane. In a separate vial, 2 ml of 2.5 mg mL^{-1} toluene solution of LC-like ligand L_1 was added to 1.512 mL of 5 mg mL^{-1} solution of L_{AZO}. Nanoparticle dispersion was then added to the ligands mixture. The mixture was left at vortex with slow (100 rpm) stirring overnight. Then, 10 mL of ethanol was added, and the mixture was centrifuged for 5 min at 6000 rpm. Finally, the supernatant was discarded, precipitated nanoparticles were redissolved in toluene, and the precipitation procedure was repeated until no unbound ligand was presented on TLC plate.

Note S5: Preparation of 12OAzo5AzoO12/NP

12OAzo5AzoO12 was the stored in a glove box under nitrogen atmosphere and protected from light. A small portion of the 12OAzo5AzoO12 compound (5 mg) was taken and dissolved in

2.5 ml of THF. This solution was stored in brown glass vials. To prepare a sample with a 9 wt% gold nanoparticle content in the 12OAzo5AzoO12 compound 5.57 µl of the Au4L_1 nanoparticle colloid (2.5 mg mL^{-1}) was placed in an empty glass vial. The solvent was evaporated. Next, 77.4 µl of 2 mg mL^{-1} 12OAzo5AzoO12 solution was added. This mixture was sonicated for 30 seconds. Directly after sonification, this mixture was dropcasted on a glass substrate for CD measurements or TEM grid or Kapton tape for microscopy or XRD measurements. Before measurements samples were heat annealed accordingly to Note S1. Samples were stored in a shaded container. To obtained samples with 5 wt%, 15 wt% and 30 wt% of Au4L_1 in 12OAzo5AzoO12 compound 3.1 µl, 9.3 µl and 18.6 µl of 2.5 mg mL^{-1} nanoparticle's dispersion should be used.

The same procedure was used to obtain samples with Au8L_1L_{AZO} NP. To obtained samples with 2.5 wt%, 5 wt% and 10 wt% of Au8L_1L_{AZO} NP in 12OAzo5AzoO12 compound 1.9 µl, 3.8 µl and 7.6 µl of 2 mg mL^{-1} nanoparticle's dispersion should be used.

Note S6: Studies of composites photoswitchability

The first step was to prepare homochiral domain on a glass substrate, according to the procedure described in Note S5.

In analogy to the purely organic films we confirmed that switching the E/Z configuration of 12OAzo5AzoO12, with UV irradiation is not perturbed. The variation of the $\pi \rightarrow \pi^*$ band intensity and CD band disappearance for sample irradiated at 80 °C was analogous to the purely organic film (Figure S34).

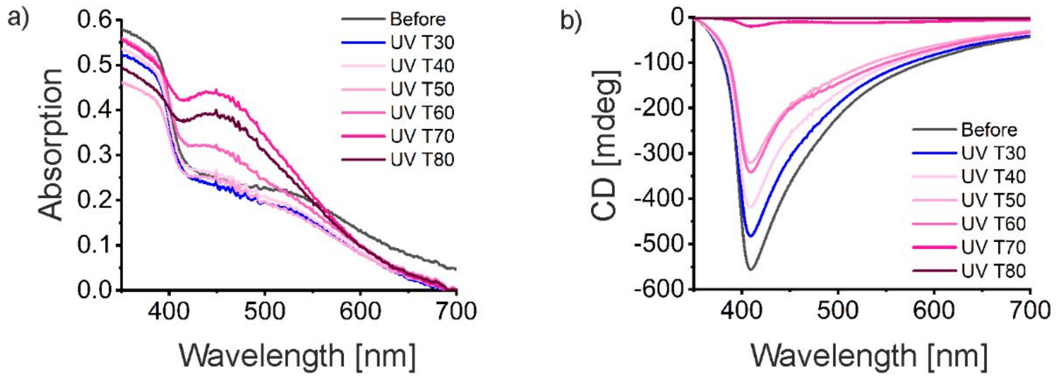

Figure S34. Optical measurements of the 9 wt.% of Au4L_1 in 12OAzo5AzoO12 thin film illuminated with UV light at different temperatures: a) UV-Vis and b) CD, respectively.

References:

(1) Bagiński, M.; Tupikowska, M.; González-Rubio, G.; Wójcik, M.; Lewandowski, W. Shaping Liquid Crystals with Gold Nanoparticles: Helical Assemblies with Tunable and Hierarchical Structures Via Thin-Film Cooperative Interactions. *Adv. Mater.* **2020**, *32* (1), 1904581. https://doi.org/10.1002/adma.201904581.

(2) Cho, E. N.; Zhitomirsky, D.; Han, G. G. D.; Liu, Y.; Grossman, J. C. Molecularly Engineered Azobenzene Derivatives for High Energy Density Solid-State Solar Thermal Fuels. *ACS Appl. Mater. Interfaces* **2017**, *9* (10), 8679–8687. https://doi.org/10.1021/acsami.6b15018.

(3) Chen, Y.; Wang, X. Novel Phase-Transfer Preparation of Monodisperse Silver and Gold Nanoparticles at Room Temperature. *Mater. Lett.* **2008**, *62* (15), 2215–2218. https://doi.org/10.1016/j.matlet.2007.11.050.

(4) Peng, S.; Lee, Y.; Wang, C.; Yin, H.; Dai, S.; Sun, S. A Facile Synthesis of Monodisperse Au Nanoparticles and Their Catalysis of CO Oxidation. *Nano Res.* **2008**, *1* (3), 229–234. https://doi.org/10.1007/s12274-008-8026-3.

(5) Wang, F.; Richards, V. N.; Shields, S. P.; Buhro, W. E. Kinetics and Mechanisms of Aggregative Nanocrystal Growth. *Chem. Mater.* **2014**, *26* (1), 5–21. https://doi.org/10.1021/cm402139r.

(6) Bagiński, M.; Pedrazo-Tardajos, A.; Altantzis, T.; Tupikowska, M.; Vetter, A.; Tomczyk, E.; Suryadharma, R. N. S.; Pawlak, M.; Andruszkiewicz, A.; Górecka, E.; Pociecha, D.; Rockstuhl, C.; Bals, S.; Lewandowski, W. Understanding and Controlling the Crystallization Process in Reconfigurable Plasmonic Superlattices. *ACS Nano* **2021**, *15* (3), 4916–4926. https://doi.org/10.1021/acsnano.0c09746.

(7) Zhang, W.; Prodanov, M. F.; Schneider, J.; Gupta, S. K.; Dudka, T.; Vashchenko, V. V.; Rogach, A. L.; Srivastava, A. K. Ligand Shell Engineering to Achieve Optimal Photoalignment of Semiconductor Quantum Rods for Liquid Crystal Displays. *Adv. Funct. Mater.* **2019**, *29* (3), 1805094. https://doi.org/10.1002/adfm.201805094.

(8) Pang, Z.; Zhang, J.; Cao, W.; Kong, X.; Peng, X. Partitioning Surface Ligands on Nanocrystals for Maximal Solubility. *Nat. Commun.* **2019**, *10* (1), 2454. https://doi.org/10.1038/s41467-019-10389-5.
